# The Legacy Effect of Mountain Pine Beetle Outbreaks on the Chemical and Anatomical Defences of Surviving Lodgepole Pine Trees

**DOI:** 10.3390/metabo14090472

**Published:** 2024-08-27

**Authors:** Gigi Baker, Shiyang Zhao, Jennifer G. Klutsch, Guncha Ishangulyyeva, Nadir Erbilgin

**Affiliations:** 1 Department of Renewable Resources, University of Alberta, Edmonton, AB T6G 2E3, Canadajennifer.klutsch@nrcan-rncan.gc.ca (J.G.K.);; 2 Natural Resources Canada, Canadian Forest Service, Northern Forestry Centre, Edmonton, AB T6G 2E3, Canada

**Keywords:** carbohydrates, bark beetle outbreaks, lodgepole pine, terpene defences, resin ducts

## Abstract

The recent mountain pine beetle outbreaks have caused widespread mortality among lodgepole pine trees in western North America, resulting in a reduced population of surviving trees. While previous studies have focused on the cascading impacts of these outbreaks on the physiology and growth of the surviving trees, there remains a need for a comprehensive study into the interactions among various physiological traits and the growth in post-outbreak stands. Specifically, the relationship between chemical (primarily terpenes) and anatomical (mainly resin ducts) defences, as well as the allocation of non-structural carbohydrates (NSCs) to support these defence modalities, is poorly understood. To address these gaps, we conducted a field survey of surviving lodgepole pine trees in post-mountain pine beetle outbreak stands in western Canada. Our retrospective analysis aimed at determining correlations between the post-outbreak concentrations of monoterpenes, diterpenes, and NSCs in the phloem and the historical resin duct characteristics and growth traits before and after the outbreak. We detected strong correlations between the post-outbreak concentrations of monoterpenes and historical resin duct characteristics, suggesting a possible link between these two defence modalities. Additionally, we found a positive relationship between the NSCs and the total concentrations of monoterpenes and diterpenes, suggesting that NSCs likely influence the production of these terpenes in lodgepole pine. Furthermore, historical tree growth patterns showed strong positive correlations with many individual monoterpenes and diterpenes. Interestingly, while surviving trees had enhanced anatomical defences after the outbreak, their growth patterns did not vary before and after the outbreak conditions. The complexity of these relationships emphasizes the dynamics of post-outbreak stand dynamics and resource allocations in lodgepole pine forests, highlighting the need for further research. These findings contribute to a broader understanding of conifer defences and their coordinated responses to forest insect outbreaks, with implications for forest management and conservation strategies.

## 1. Introduction

Periodic outbreaks of bark beetles (Coleoptera: Curculionidae, Scolytinae) have caused extensive tree mortality, significantly altering forest structure and function [1,2,3] and shifting affected areas from carbon sinks to carbon sources [4,5,6]. For instance, recent outbreaks of the mountain pine beetle (MPB, *Dendroctonus ponderosae* Hopkins) have devastated millions of hectares of mature lodgepole pine (*Pinus contorta*) forests in western North America [1,2]. Despite this widespread mortality, certain individual trees—pine and non-pine species—have survived these outbreaks [7,8,9]. These surviving trees are crucial for maintaining ecological resilience within bark beetle-affected forests and may serve as a genetic reservoir of traits that confer resistance to future outbreaks, thereby contributing to the overall health and diversity of forest ecosystems [10,11,12,13,14]. Given their importance, numerous studies have reported on the impact of bark beetle outbreaks on these surviving trees, focusing on their post-outbreak defences (chemical or anatomical) [7,8,9] and post-outbreak growing conditions [10,11,12,13,14]. While several studies have separately examined the impact of bark beetle outbreaks on anatomical and chemical defence mechanisms of the surviving trees, e.g., [7,8,9], few have explored the complex relationship between these defence modalities in the post-outbreak stands. This study aims to retrospectively examine the relationship between the constitutive terpenes in the current year’s phloem of surviving pine trees and the historical resin duct characteristics of the same trees before and after the MPB outbreak in lodgepole pine stands affected by the outbreak.

Interactions between conifers and bark beetles represent well-studied systems, integrating molecular, chemical, and ecological perspectives to elucidate the outcomes of these interactions [15,16,17]. Bark beetles feed, mate, and lay eggs within the subcortical tissues (phloem/cambial zone) of mature trees, making them prominent agents of mortality in coniferous forests [18]. When confronted with bark beetle attacks, conifer trees employ a multifaceted defence strategy, involving both defence compounds and anatomical structures [18,19,20,21,22,23,24]. Resin, a mixture of terpenes, serve as the primary defence against bark beetles for conifers. Monoterpenes and diterpenes, in particular, have received more attention due to their biological effects on bark beetles and their fungal associates [25,26,27]. Pine phloem, the feeding site for bark beetles, contains large quantities of monoterpenes and diterpenes, which are expressed constitutively and are induced [21,28]. The production of these terpenes is supported in part by non-structural carbohydrates (NSCs), such as soluble sugars and starch [29,30,31,32,33]. These NSCs are vital for various physiological and metabolic processes in trees [31,32,33]. They also serve as substrates for secondary metabolite production [33]. Changes in carbohydrate production can impact both the storage and production of secondary metabolites, such as terpenes [31,32]. While some studies report a positive association between terpenes and NSCs in tree phloem [29], others do not [34].

Anatomical defences of conifers primarily involve the synthesis and storage of resin in specialized structures known as resin cells and resin ducts [18,20]. These structures are interconnected axially and radially. Resin ducts are distributed throughout the primary and secondary xylem as well as the cortical tissues. Resin accumulated within these structures is transported to the point of bark beetle attacks. Consequently, the morphology of xylem resin ducts is often associated with the quantity of resin flow in response to wounds in the phloem [35,36,37,38,39,40]. In some cases, resin ducts are linked to tree survival against bark beetles, with larger duct sizes and higher duct numbers showing stronger relationships with survival [8,41,42,43].

Our earlier investigations demonstrated distinct anatomical defence traits in surviving trees following MPB outbreaks compared to those killed by MPBs [8]. Consequently, the surviving trees contained higher concentrations of monoterpenes [7]. However, the interaction between these chemical and anatomical defences remains unknown within the lodgepole pine system. Therefore, our primary objective is to retrospectively investigate whether the constitutive concentrations of post-outbreak monoterpenes and diterpenes in the phloem of surviving pine trees correlate with the historical resin duct characteristics, radial growth, and tree growth traits (age and size) before and after MPB outbreaks in post-outbreak lodgepole pine stands. Additionally, we explored potential relationships between defence traits (chemical or anatomical) and NSCs in the phloem and any trade-offs between monoterpene and diterpene defences following the MPB outbreak. This study aims to shed light on the complex relationship between various defence modalities and tree characteristics amidst the MPB outbreak, providing valuable insights into the legacy impact of bark beetles on host trees.

## 2. Materials and Methods

### 2.1. Site Selection

In July 2020, we identified five lodgepole pine-dominated forests in Jasper National Park, Alberta (Canada). Lodgepole pine constituted 45–59% of the total live and dead tree density (height > 1.3 m) in sites 1–3 and 100% in sites 4 and 5 (Table S1). Douglas-fir (*Pseudotsuga menziesii*), white spruce (*Picea glauca*), and aspen (*Populus tremuloides*) were the other tree species in these sites. All sites had experienced a recent MPB outbreak, as documented in historical records provided by Parks Canada. The outbreaks occurred in sites 1–3 in 2015 and in sites 4 and 5 in 2014. The sites were located at least 500 m apart. We gathered data on the on-site slope using a clinometer and aspect using a compass. Tree density at a site was calculated using an angle gauge with a basal area factor of 4.6 metric (20 standard). The tree species, health status (alive or killed by MPBs), and diameter at breast height (DBH, 1.3 m height) were measured for all trees within the plot (Table S1). Trees killed by MPBs were identified through characteristic pitch tubes, brood emergence holes on the tree stems, blue stains, and oviposition (maternal) galleries under the tree bark [7]. Conversely, the healthy lodgepole pines exhibited no visible symptoms of insect or pathogen attacks.

### 2.2. Sample Collection

We selected 12 pairs of beetle-killed and healthy lodgepole pine trees in each site, with the DBH ranging from 18 to 22 cm (*n* = 60 trees per category). We obtained two phloem samples using a leather hollow punch that was 1.9 cm in diameter from each of the four cardinal directions at 1.3 m from ground on healthy trees (8 samples per tree). The punch was cleaned using 70% ethanol before each sampling. The samples were wrapped in labelled aluminium foil and immediately placed into liquid nitrogen. The samples were stored on dry ice during transportation from the field to the laboratory and put in a −40 °C freezer until chemical analysis.

Furthermore, we obtained one increment core with a diameter of 5 mm and one with a diameter of 12 mm at 1 m above the ground from the north and south aspects of each health tree. The core that was 5 mm in diameter was drilled to the pith of the tree and used to determine its age. The cores with a 12 mm diameter contained at least 20 years of annual rings, dating back from the sampling year, which were used to determine the anatomical defence and growth characteristics of trees in the last 15 years. The cores were stored in plastic drinking straws. To determine the year of the outbreak in each site, we took a wedge from the south-facing aspect of each beetle-killed tree at the DBH. The wedges contained at least 20 years of growth, counting back from the year of death.

### 2.3. Chemical Analysis

After removing the outer bark from the phloem samples, we ground the samples in liquid N with a mini bead beater at 2200 rpm for three 20 s intervals. The eight samples from each tree were pooled together before analysis. The samples from each tree were divided into three groups for the following chemical analyses: monoterpenes (100 mg), diterpenes (30 mg), and NSCs (30 mg). All these analyses followed the protocols reported by Cale et al. [44].

#### 2.3.1. Monoterpene Analysis

At room temperature, 0.5 mL of hexane with pentadecane (0.004%) (internal standard) was mixed with 100 mg of the sample. The mixture was vortexed for 30 s, sonicated for 10 min, and then centrifuged for 15 min at 18,213 g at 4 °C. This process was repeated twice for each sample. The extract was transferred to a 2 mL GC (gas chromatograph) vial and stored at −40 °C until analysis. Extracts were analysed with a GC-MS (GC 7890A, Mass Spectrometry (MS) 5975C, Agilent Tech., Santa Clara, CA, USA) fitted with an Agilent HP-Chiral 20B column (30 m × 0.25 mm ID × 0.25 µm film). The carrier gas was helium at a flow rate of 1.0 mL min^−1^. The temperature began at 50 °C and increased to 72 °C at a rate of 75 °C min^−1^, where it was held for 0.5 min, then increased at 30 °C min^−1^ to 90 °C and held for 2 min. It was then increased to 95 °C at 3 °C min^−1^ and held for 1 min, then increased to 100 °C at 5 °C min^−1^ and held for 0.5 min, then to 150 °C at 8 °C min^−1^, and finally, to 175 °C at 15 °C min^−1^ and held for 1.5 min. The total run time for each sample was 23.6 min. We identified the hexane-extractable compounds, mostly monoterpenes, one phenylpropanoid (4-allyanisole), and one terpene derivative and its acetate ester (borneol and bornyl acetate) (hereafter, monoterpenes), by comparing the mass spectra and retention times to those of known standards. 

Monoterpenes were quantified using standard curves from four dilutions prepared from analytical standards of (+)-limonene (chemical purity, 99%); (−)-limonene (99%); α-terpinene (95%); (+)-α-pinene (98.5%); (−)-α-pinene (98%); myrcene (94%); 3-carene (98.5%); (+)-β-pinene (98%); (−)-β-pinene (99%); β-phellandrene (90%); (+)-camphene (90%); γ-terpinene (97%); terpinolene (90%); 4-allylanisole (98.5%); R-(+)-pulegone (97%); bornyl acetate (97%); (−)-borneol (99%); linalool (98%); ocimene (90%); and α-terpineol (90%). Concentrations were reported as “µg mg^−1^ of dry weight (DW)” phloem tissue.

#### 2.3.2. Diterpene Analysis

Diterpenes were extracted from 30 mg of lyophilized ground tissue in 1 mL of methanol. The mixture was vortexed for 30 s then centrifuged for 10 min at 18,213 rcf at 4 °C. The supernatant was transferred to 2 mL GC vials and kept at −40 °C until analysis. Diterpene acid extractions were analysed using an Ultra-High Performance Liquid Chromatograph (UHPLC, 1290 Infinity, Agilent Tech.) fitted with an InfinityLab Poroshell 120 EC-C18 column (2.1 × 150 mm 1.9 µm, Agilent Tech.) and a Diode Array Detector (UV/Vis, 1290 DAD, Agilent Tech.). Gradient analyses were performed with a binary solvent system: 1.7% *v*/*v* acetic acid with ultra-pure water (A) and 100% methanol (HPLC grade) (B) with a flow rate of 0.3 mL min^−1^. A 5 µL volume was used. The system started at 75% B for 1 min, then increased to 85% B over 9 min, held for 2 min, decreased to 75% B over 2 min, and held at 3 min. The total run time was 17 min. Diterpene resin acids were quantified using wavelengths of 240, 268, and 282 nm, as adapted from a multiwavelength detection approach from Kersten et al. [45]. Standard curves calculated from dilutions prepared of pimaric acid (80%), abietic acid (95%), dehydroabietic acid (99%), isopimaric acid (99%), levopimaric acid (95%), neoabietic acid (99%), palustric acid (92%), and sandarocopimaric acid (90%) were used to quantify diterpene acids. Pimaric and isopimaric acids were listed together due to coelution with abietic acid and cannot be quantified individually. Concentrations were reported as “µg mg^−1^ of DW” phloem tissue.

#### 2.3.3. Non-Structural Carbohydrate (Soluble Sugars and Starch) Analyses

All three soluble sugars (glucose, fructose, and sucrose) were extracted from 30 mg of the lyophilized and ground phloem tissue. The sample was mixed with 1.3 mL of ultra-pure water in a 2 mL tube. A marble was placed in the opening of the tube. Tubes were placed above a boiling pot of water and enclosed in steam for 1 h. A 0.5 mL aliquot was collected and centrifuged. Then, 0.4 mL of a supernatant was placed in a new 2 mL tube with 1.0 mL of HPLC-grade methanol. The extracts were then incubated at room temperature for one h. Then, 0.5 mL of the extract was transferred to 2 mL GC vials and kept at −40 °C until analysis.

Glucose, fructose, and sucrose were analysed with the UHPLC system that was fitted with an Infinity Lab Poroshell 120 HILIC-Z column (2.1 × 100 mm 2.7 µm, Agilent Tech., Santa Clara, CA, USA) as well as with an Evaporative Light Scattering Detector (ELSD, 1290 ELSD II, Agilent Tech., Santa Clara, CA, USA). A gradient analysis was performed using a binary solvent system of 0.034% *v*/*v* ammonium hydroxide to buffer ultra-pure water (A) as well as acetonitrile (HPLC grade), also buffered with 0.034% *v*/*v* ammonium hydroxide (B). The flow rate was set to 0.2 mL min^−1^. A 4 µL injection volume was used. The solvent system started at 90% B for 2 min then decreased to 75% B over 3.5 min, where it was held for 1 min. It was then increased to 90% B over 2 min and held there for 0.5 min. The total run time was 9 min. The ELSD settings were set so that the nebulizer temperature was 25 °C, the evaporator tube temperature was 60 °C, and the gas flow rate was 1.40 SLM. Dilutions prepared from analytical standards of glucose (99%), fructose (99%), and sucrose (99.55%) were used to calculate standard curves to quantify soluble sugars. 

For starch extraction, 25 mg of the lyophilized ground phloem tissue was used. Enzymatic digestions were used to convert starch into gluconate-6-phosphate, as described in Cale et al. [44]. As with the soluble sugar extraction, samples were held in steam for 1 hr. Samples were then vortexed for 30 s. A 0.4 mL aliquot was transferred to a new 2 mL tube with 0.4 mL of an alpha-amylase solution of 75 g of an enzyme (Sigma-Aldrich, St. Louis, MO, USA) in 100 mL of ultra-pure water. This enzyme converts starch to large soluble sugars (e.g., maltose). The tubes were vortexed immediately for 30 sec and then incubated in a 50 °C bath for 16 h. Following the incubation, tubes were removed from the water bath and then inverted twice. The extracts were then centrifuged at 18,213 rcf for 15 min, which removed undigested glucose-containing polymers (e.g., hemicellulose). After this, 0.4 mL of the supernatant was transferred to a new 2 mL tube containing 0.4 mg of an amyloglucosidase solution consisting of 3 g of an enzyme (Sigma-Aldrich, St. Louis, MO, USA) in a 60 mL sodium acetate buffer (0.1 M, pH 4.5). This enzyme converts maltose and other large sugars to glucose. The tubes were incubated in a 50 °C bath for 16 h. Tubes were removed from the water bath and inverted twice. The extracts were centrifuged at 18,213 rcf for 15 min. Then, 0.4 mL of the extracts were transferred to 2 mL GC vials and stored at −40 °C until analysis. 

We pipetted 0.020 mL of the starch extract into a 96-well plate. Next, 0.2 mL of glucose hexokinase (Sigma Aldrich)—isomerase (phosphoglucose isomerase, Sigma Aldrich)—was added to each well. This converted starch aliquots into gluconate-6-phosphate, as described by Cale et al. [44]. The well plate was shaken at room temperature for 45 min on an orbital shaker. Then, the amount of gluconate-6-phosphate was measured at an absorbance of 340 nm using the Synergy Microplate Reader H1 (BioTek, Winooski, VT, USA).

The starch concentration was quantified using two calibration curves: the first to estimate the glucose concentration from the amount of gluconate-6-phosphate measured and the second to estimate the starch concentration using the estimated glucose concentration. To do this, the sample concentration of the total glucose was standardized by sample dry weights and extraction volumes after the first calibration curve. Previously determined concentrations of soluble glucose and fructose (hexoses) were subtracted from the estimated total glucose concentration. A conversion factor was applied to account for potential quantification differences. The second calibration curve used dilutions of pure potato starch standards processed with the same enzymatic digestions as described above. The potential matrix effects were calculated to adjust the final concentrations accordingly.

### 2.4. Anatomical Defence and Growth Characteristics

Core and wedge samples were glued to wooden mounts and dried for two weeks at room temperature. Samples were then sanded with belt and hand sanders using progressively finer sandpapers. Samples were then scanned at 1200 dpi to create high-resolution digital images. The annual growth (mm) was measured from the pith to last year’s annual ring using ImageJ for all core samples [8]. Calendar years were assigned to each annual ring by visual cross-dating ring characteristics, such as the width ratio and the colour contrast between earlywood and latewood per ring. This information was used for wedges to determine the year of death of trees killed by MPBs. The earliest death recorded was considered the starting year of the outbreak in a particular site. Among the five sites, two sites experienced an outbreak in 2014, and the remaining three sites had an MPB outbreak in 2015. Therefore, the healthy trees were separated into two outbreak years: 2014 (*n* = 24) and 2015 (*n* = 36).

All samples for resin ducts were examined using ImageJ within a fixed sampling width of 10 mm [8]. To analyse the resin ducts, the resin duct production (number of resin ducts per 10 mm width per year on a ring [no. year^−1^]), resin duct size (mean size of resin ducts per 10 mm width per year on a ring [mm^2^ year^−1^]), total resin duct area (sum of resin duct area per 10 mm width per year on a ring [mm^2^ year^−1^]), resin duct density (total number of resin ducts per year divided by the ring area (10 mm * ring width) for a given year [no. mm^2^ year^−1^], and relative resin duct area (percent area occupied by resin ducts per year within a ring area for a given year [% year^−1^]) were calculated for each core that had a diameter of 12 mm [12,43]. The ring width (mm year^−1^) and basal area increment (BAI) (mm^2^ year^−1^) were used to represent tree radial growths. Tree radii and ring width data were used to calculate the BAI, assuming that tree rings are concentric circles. Tree radii were calculated by dividing the tree DBH by two.

### 2.5. Data Analysis

To study the long-term relationship between the growth and defence mechanisms in healthy pine trees, we analysed the annual growth and resin duct characteristics across three distinct periods: two pre-outbreak periods (5 years and 10 years before the outbreak) and one post-outbreak (from outbreak onset until 2019, when the last annual ring was formed at the time of sampling) period.

We conducted Pearson correlation tests to examine correlations between terpenes (21 monoterpenes, six diterpene resin acids, total monoterpenes, and total diterpenes); NSCs (three soluble sugars, starch, and total NSCs); and five anatomical defence traits and two radial growth characteristics. The analysis was performed using R (Version 4.0.5) [46], using the *Hmisc* R package [47]. We selected the BAI to represent tree radial growths, as it strongly correlates with the ring width (Pearson correlation coefficient was > 0.96 in both the 2014 and 2015 outbreak years). In the analyses, we considered the overall as well as the total and individual concentrations of the constitutive monoterpenes and diterpenes.

#### 2.5.1. Gradient Analysis

We employed an indirect gradient analysis to examine the impact of resin duct characteristics, BAIs, tree ages, and DBHs on the overall monoterpenes, diterpenes, and NSCs before and after outbreaks. We utilized non-linear multidimensional scaling (NMDS) with the Bray–Curtis dissimilarity to ordinate monoterpenes, diterpenes, and NSCs using the vegan R package [48]. The NMDS results were saved as RData files to ensure reproducibility. Subsequently, we projected resin duct characteristics from pre- and post-outbreak periods onto NMDS axes as environmental gradients and visualized these relationships using ordination triplots created with the ggplot2 R package [49]. We followed the same steps when incorporating the radial growth characteristics from pre- and post-outbreak periods and the tree growth traits (age and DBH) into the NMDS axes for the 2014 and 2015 outbreak years.

#### 2.5.2. Linear Mixed-Effect Models

To identify relationships among defence and growth characteristics, we employed linear mixed-effect models, treating the site as a random effect and using the R package lme4 [50]. We visually assessed model assumptions, including the normality and homogeneity of residuals, applying transformations and rescaling as needed. The resin duct density and the percent resin duct area were standardized per 1 mm^2^ and accounted for the BAI prior to calculation. Furthermore, we investigated the relationships between the total monoterpenes or diterpenes and tree growth traits using linear mixed-effect models. When we observed a statistically significant relationship, we conducted further linear mixed-effect models with the site as a random effect to examine the specific relationship between individual monoterpenes or diterpenes and a particular predictor. In all cases, significance was determined at α = 0.05.

#### 2.5.3. Comparative Analyses

We compared anatomical defences and radial growth responses across pre- and post-outbreak periods within the 2014 and 2015 outbreak years using linear mixed-effect models. The variability across sites was accounted for by nesting trees within sites. Furthermore, we examined the differences in tree age and DBH between the 2014 and 2015 outbreak years using linear mixed-effect models, where the site was considered a random effect to address any unexplained variances among the trees. 

## 3. Results

### 3.1. The Historical Resin Duct Characteristics Affect the Current Year’s Terpenes

#### 3.1.1. Monoterpenes

**Figure 1:**
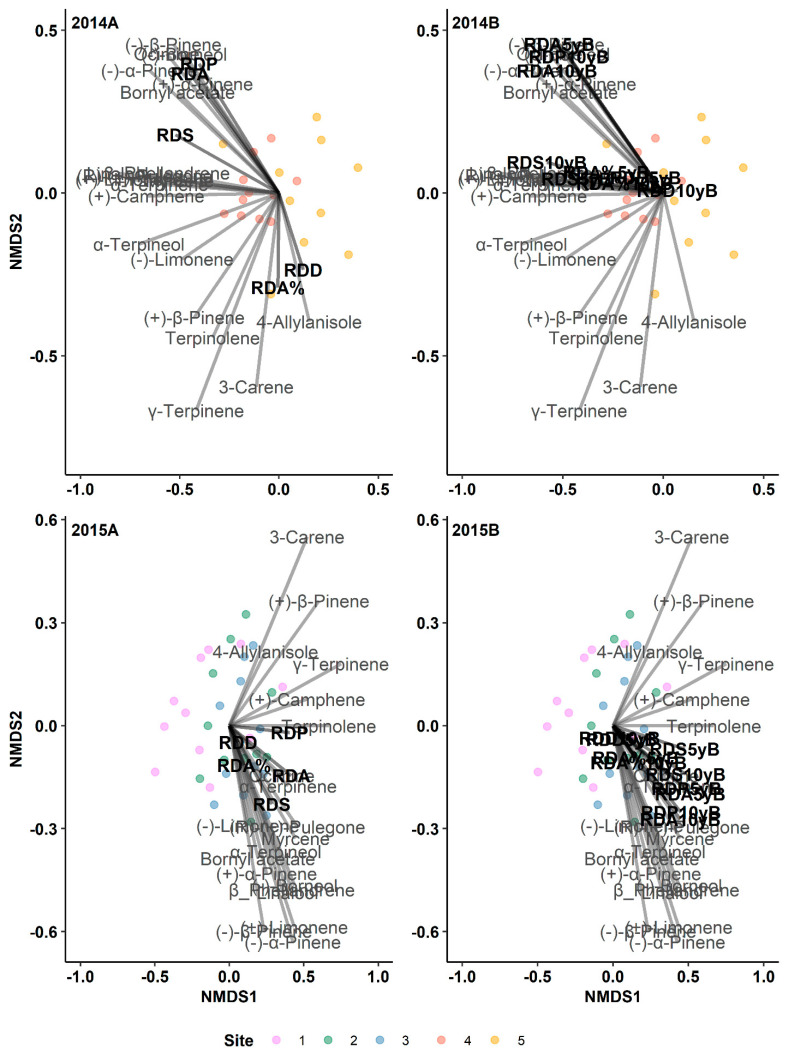
Results of indirect gradient analysis by NMDS with Bray–Curtis dissimilarity showing the relationship between monoterpenes and the annual resin duct characteristics of lodgepole pine trees in post-outbreak (**2014A**, **2015A**) and 5-year and 10-year pre-outbreak (**2014B**, **2015B**) periods in Jasper National Park (Alberta, Canada). RDP: Resin duct production; RDA: Total resin duct area; RDS: Individual resin duct size; RDD: Resin duct density; RDA%: Relative resin duct area; 5yB: 5-year pre-outbreak; 10yB: 10-year pre-outbreak.

We observed several significant relationships between some of the resin duct characteristics and overall monoterpenes across both the 2014 and 2015 outbreak years (Figure 1, Table 1). In the 2014 outbreak, the relationship between the total monoterpenes and resin duct production and the total resin duct area were significant during all three periods. The resin duct size was also positively correlated with the total monoterpenes in the post-outbreak and 10-year post-outbreak period. In the 2015 outbreak, the total resin duct area and the resin duct size displayed significant positive relationships with the total monoterpenes in all three time periods. A further testing of the relationship between individual monoterpenes and three resin duct predictors (resin duct production, total resin duct area, and individual resin duct size) yielded significant results. The individual monoterpene concentrations across the sites are shown in Table S2. All three resin duct characteristics exhibited positive trends with several monoterpenes in the 2014 and 2015 outbreak years (Table 2). In the 2014 outbreak, we observed significant relationships between the resin duct characteristics and 16 monoterpenes, while 15 monoterpenes displayed significant relationships with the resin duct characteristics in the 2015 outbreak. Among the monoterpenes examined, several had significant relationships with at least one of the resin duct characteristics. Notably, (+)-limonene, (−)-α-pinene, (+)-β-pinene, β-phellandrene, myrcene, (+)-camphene, (−)-borneol, and linalool correlated with most of the resin duct characteristics in both outbreak years (Table 2). Additionally, α-terpinene, (+)-α-pinene, 3-carene, γ-terpinene, terpinolene, (R)-(+)-pulegone, bornyl acetate, ocimene, and α-terpineol also exhibited significant relationships with at least one of the resin duct characteristics.

#### 3.1.2. Diterpenes

**Figure 2:**
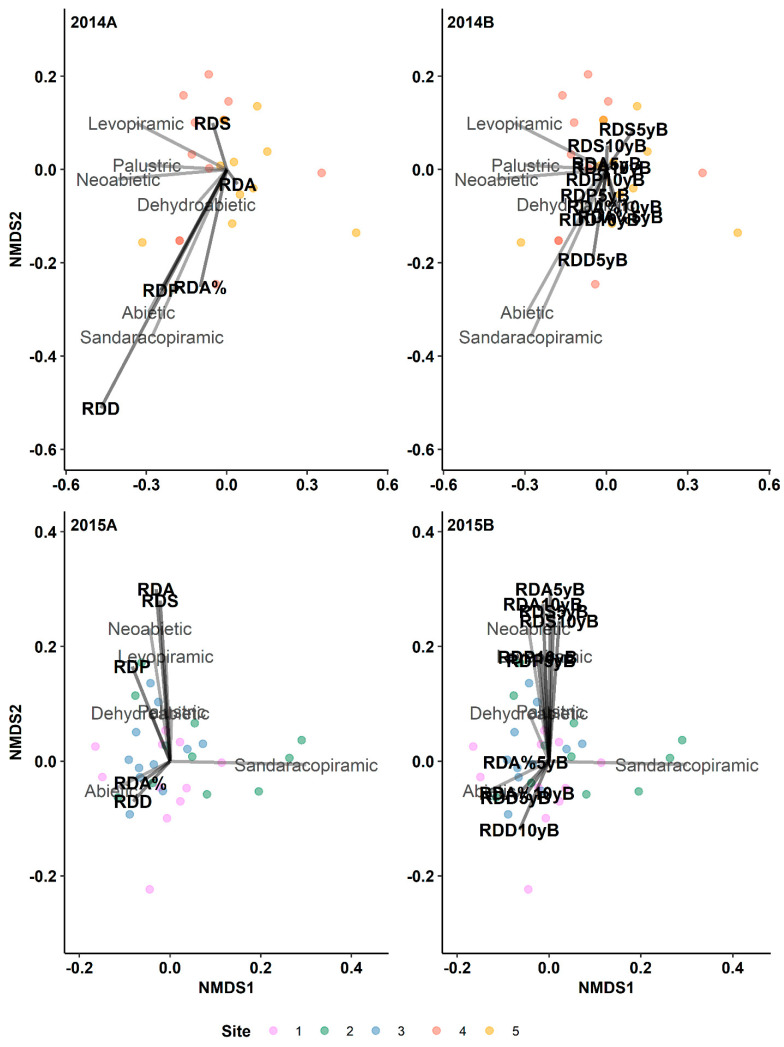
Results of indirect gradient analysis by NMDS with Bray–Curtis dissimilarity showing the relationship between diterpenes and the annual resin duct characteristics of lodgepole pine trees in post-outbreak (**2014A**, **2015A**) and 5-yeaer and 10-year pre-outbreak (**2014B**, **2015B**) periods in Jasper National Park (Alberta, Canada). RDP: Resin duct production; RDA: Total resin duct area; RDS: Individual resin duct size; RDD: Resin duct density; RDA%: Relative resin duct area; 5yB: 5-year pre-outbreak; 10yB: 10-year pre-outbreak.

We did not observe any relationship between any of the resin duct characteristics and diterpene profiles or total diterpenes or individual diterpenes in either the pre- or post-outbreak periods within the 2014 and 2015 outbreak years, suggesting no association between phloem diterpenes and xylem anatomical defences (Figure 2).

### 3.2. NSCs Affected the Total Terpenes in the 2015 Outbreak but Not in the 2014 Outbreak

In the 2014 and 2015 outbreaks, there were no significant relationships between any of the NSCs and overall monoterpenes (Figure S1 and Table S3) or diterpenes (Figure S2 and Table S3). However, when we tested the relationship between the total NSCs and the total monoterpenes or total diterpenes within the 2015 outbreak, the NSCs were significantly and positively related to the total diterpenes (coefficient = 0.701 and *p* = 0.019) and the total monoterpenes (coefficient = 59.25 and *p* = 0.048). We did not observe any significant relationships in the 2014 outbreak year.

### 3.3. The Radial Growth Characteristics Affect Terpene Production

**Figure 3:**
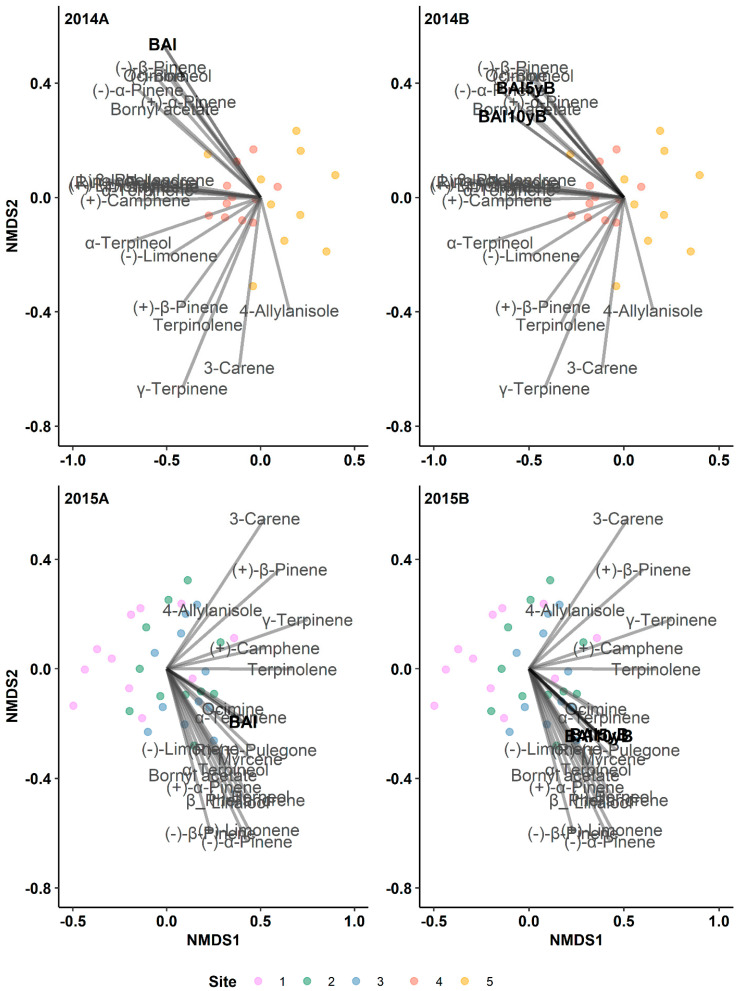
Results of indirect gradient analysis by NMDS with Bray–Curtis dissimilarity showing the relationship between monoterpenes and the annual basal area increment (BAI, mm^2^ year^−1^) of lodgepole pine trees in post-outbreak (**2014A**, **2015B**) and 5-year and 10-year pre-outbreak (**2014B**, **2015B**) periods in Jasper National Park (Alberta, Canada). 5yB: 5-year pre-outbreak; 10yB: 10-year pre-outbreak.

**Figure 4:**
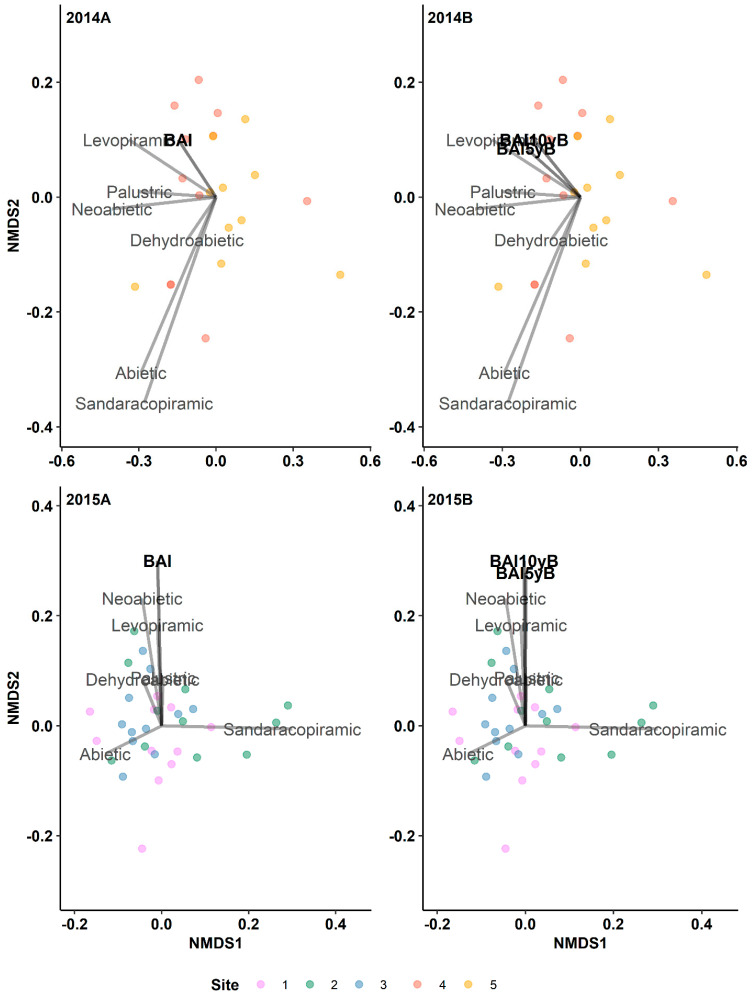
Results of indirect gradient analysis by NMDS with Bray–Curtis dissimilarity showing the relationship between diterpenes and the annual basal area increment (BAI, mm^2^ year^−1^) of lodgepole pine trees in post-outbreak (**2014A**, **2015B**) and 5-year and 10-year pre-outbreak (**2014B**, **2015B**) periods in Jasper National Park (Alberta, Canada). 5yB: 5-year pre-outbreak; 10yB: 10-year pre-outbreak.

Due to the strong correlation between the BAI and the annual radial growth rate (ring width), we opted to use the BAI as a representation of the radial tree growth to account for the tree size. Overall, the monoterpenes are strongly associated with the BAI across all pre- and post-outbreak periods (2014 outbreak: r^2^ values range from 0.414 to 0.516 and *p*-values range from 0.01 to 0.001; 2015 outbreak: r^2^ values range from 0.194 to 0.202 and *p*-values range from 0.047 to 0.033; Figure 3). Conversely, the relationship between the diterpenes and BAI was marginally significant during the 10-year pre-outbreak period in the 2015 outbreak (*p* = 0.05; Figure 4 and Table S4). 

Furthermore, we detected a positive relationship between the total monoterpenes and BAI during pre- and post-outbreak periods in both the 2014 and 2015 outbreaks (Table 3). The BAI also exhibited a significant and positive relationship with the total diterpenes across all pre- and post-outbreak periods in the 2015 outbreak (Table 3); however, in the 2014 outbreak, we did not detect any significant relationship between the BAI and the total diterpenes.

Our analysis revealed strong positive relationships between individual monoterpenes and BAIs (Table 4). The BAI had a significantly positive relationship with 65% of the individual monoterpenes in all pre-and post-outbreak periods in the 2014 outbreak and 50% of individual monoterpenes in the same periods in the 2015 outbreak. Specifically, (+)-limonene, (−)-α-pinene, myrcene, (+)-β-pinene, β-phellandrene, (+)-camphene, and linalool exhibited consistent positive relationships with the BAI across all time periods in both outbreak years. However, this relationship appears to be stronger in the 2014 outbreak. Additionally, within the 5-year and 10-year pre-outbreak periods, the relationship between the individual monoterpenes and BAI was more pronounced.

The analysis revealed a strong positive relationship between individual diterpenes and BAIs across all pre- and post-outbreak periods, specifically within the 2015 outbreak (Table 4). This relationship extended to various diterpenes, including dehydroabietic, levopimaric, palustric, neoabietic, and abietic acids. In contrast, no significant relationship was observed between the individual diterpenes and BAI within the 2014 outbreak.

### 3.4. Tree Age, but Not DBH, Affects Terpene Production

**Figure 5:**
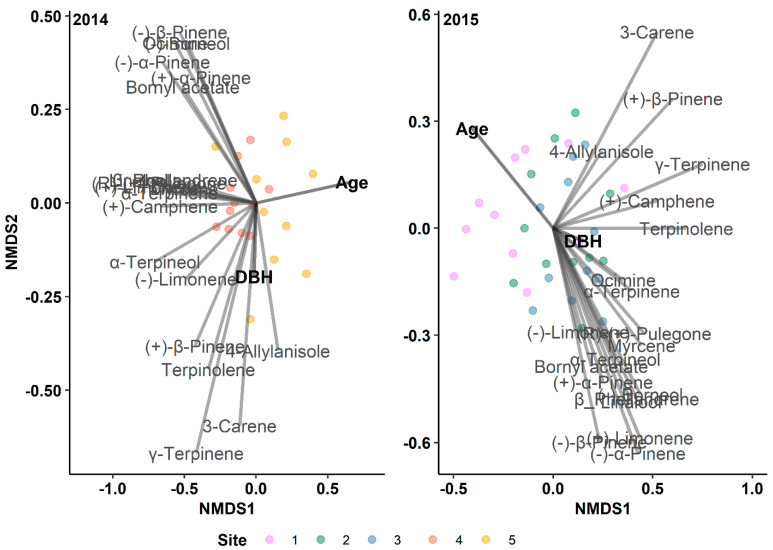
Results of indirect gradient analysis by NMDS with Bray–Curtis dissimilarity showing the relationship between monoterpenes and diameter at breast height (DBH) and age of lodgepole pine trees in Jasper National Park (Alberta, Canada) for different outbreak years.

Even though the trees sampled had similar DBH values in 2014 and 2015 (2014: 22.19 ± 0.59 cm; 2015: 20.76 ± 0.32 cm), there was a notable difference in the mean age of the trees between the two outbreak years (2014: 64.27 ± 3.01; 2015: 80.42 ± 3.85; *p* = 0.005). While the DBH did not correlate with the monoterpenes in either outbreak year, tree age exhibited a significant relationship with the monoterpenes in both the 2014 and 2015 outbreaks (P_2014_ = 0.001 and P_2015 =_ 0.017; Figure 5 and Table S5). The tree age negatively correlated with the total (P_2014_ = 0.043 and *t*-value: −2.2; P_2015_ = 0.009 and *t*-value: −2.8; Figure 5) and individual (Table 5 and Figure 5) monoterpenes. The tree age was linked to 45% of the individual monoterpenes in the 2014 outbreak and 40% in the 2015 outbreak. Briefly, (+)-limonene, (−)-α-pinene, γ-terpinene, and linalool strongly correlated with tree age in both outbreak years.

The analysis indicated that the tree DBH and age had no significant effects on the diterpenes. Additionally, the individual diterpenes did not exhibit any relationships with the DBH or age (Figure S3) within the 2014 or 2015 outbreak years. Given these findings, further tests examining the relationships between individual diterpenes and tree ages or DBHs were not pursued, as no significant associations were detected.

### 3.5. There Was No Relationship between NSCs and Anatomical Defence Traits, Radial Growth Rate, and Tree Growth Traits

The results from the indirect gradient analysis indicated that neither the anatomical defences (Figure S4 and Table S6) nor the radial increment growth rate (BAI; Figure S5 and Table S4) within the xylem had any substantial relationships with NSCs in the phloem for both the 2014 and 2015 outbreak years. Similarly, non-significant results were obtained when investigating the relationship between the total NSCs and each resin duct characteristic (Table 1) and BAI (Table 3) for both the 2014 and 2015 outbreaks. Additionally, neither tree age nor the DBH appeared to impact the NSC profile, individual NSCs, or total NSCs (Figure S6). 

### 3.6. There Is No Trade-Off between Monoterpenes and Diterpenes

We found no significant relationships between the monoterpenes and diterpenes in the 2014 or 2015 outbreaks (Figure S7 and Table S7). Additionally, the relationship between the total monoterpenes and total diterpenes was insignificant in this study.

### 3.7. Resin Duct Characteristics Post-Outbreak Varied with Growth Responses across Time Periods

**Figure 6:**
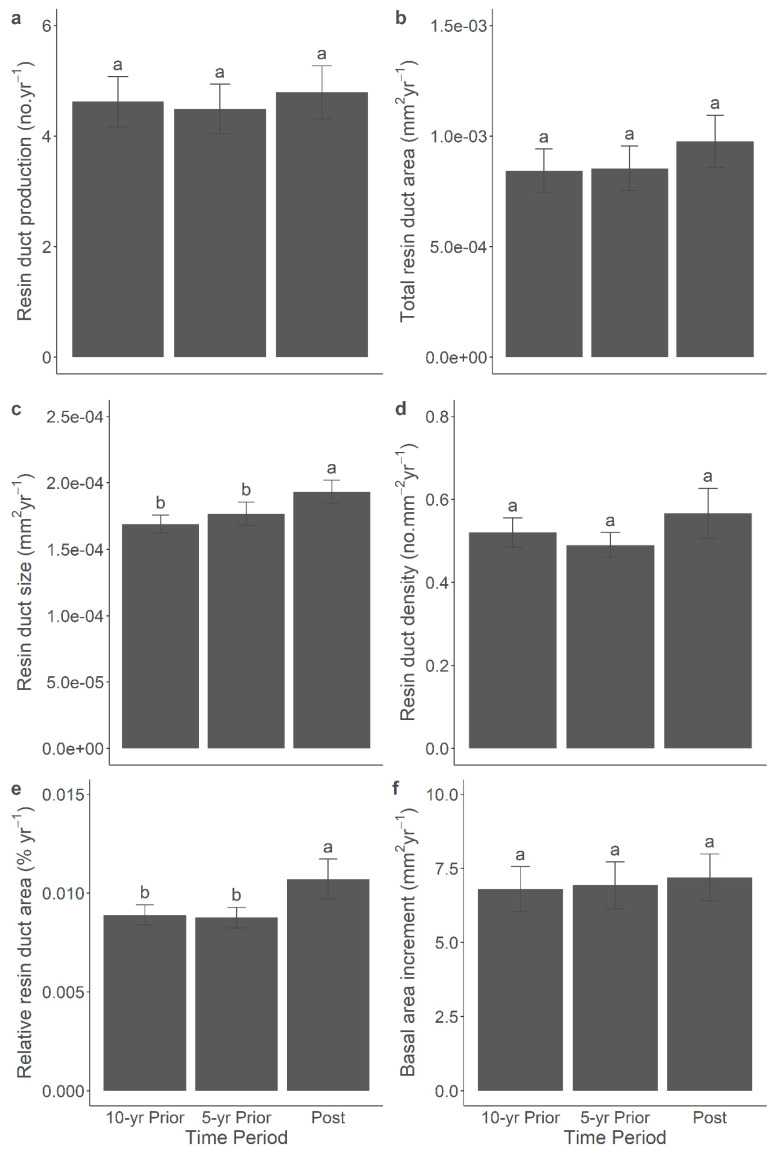
Mean (±SE) resin duct production (**a**), total resin duct area (**b**), resin duct size (**c**), resin duct density (**d**), relative resin duct area (**e**), and basal area increment (**f**) of lodgepole pine trees in 5-year and 10-year prior- and post-outbreak periods in the 2014 outbreak. Significant differences among periods were indicated by different letters.

**Figure 7:**
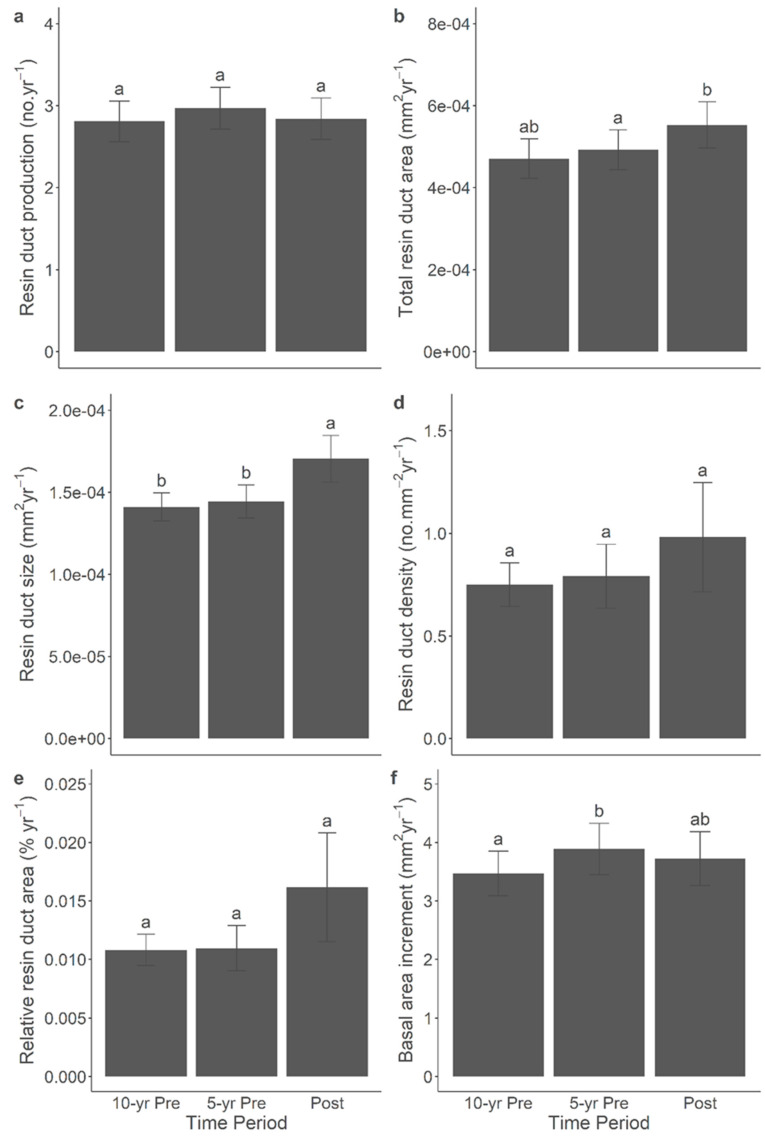
Mean (±SE) resin duct production (**a**), total resin duct area (**b**), resin duct size (**c**), resin duct density (**d**), relative resin duct area (**e**), and basal area increment (**f**) of lodgepole pine trees in 5-year and 10-year prior- and post-outbreak periods in the 2015 outbreak. Significant differences among periods were indicated by different letters.

The correlation between the anatomical defence and growth characteristics was significant across all time periods. Additional comparisons of the anatomical defences and the BAI among the post-outbreak and the 5-year pre-outbreak and 10-year pre-outbreak periods yielded significant results within both the 2014 (Figure 6) and 2015 (Figure 7) outbreaks. Overall, in both outbreaks, the resin duct characteristics exhibited a greater size and relative area during the post-outbreak period compared to the 5-year and 10-year pre-outbreak periods. For instance, the annual resin duct size following the 2014 or 2015 outbreaks was larger by approximately 10–20% compared to both pre-outbreak periods. In the 2014 outbreak, the relative resin duct area after the outbreak was about 20% larger than in both the 5-year and 10-year pre-outbreak periods, even though the BAI did not exhibit a significant response to the outbreak. In the 2015 outbreak, which comprised older pine trees, the BAI during the 5-year pre-outbreak period was 12% larger than in the 10-year pre-outbreak period. However, no significant growth release was observed following the 2015 outbreak. Moreover, the total resin duct area during the post-outbreak period was 12% larger than that during the 5-year pre-outbreak period but was similar to that observed in the 10-year pre-outbreak period. Notably, the resin duct production, density, and size displayed stronger responses following the 2014 and 2015 outbreaks than before. It is important to mention that including the site as a random effect in the linear mixed-effect models represented relatively high variance, which might contribute to the variations observed in the terpenes and NSCs.

## 4. Discussions

We conducted a retrospective analysis of the constitutive monoterpenes and diterpenes, NSCs, and their interactions with anatomical defences and growth characteristics in surviving lodgepole pine trees in post-MPB-outbreak stands. We observed a strong correlation between post-outbreak concentrations of monoterpenes in the phloem and the historical resin duct characteristics in the xylem, along with a positive association between NSCs and the total monoterpenes and diterpenes. Despite finding no evidence of growth release (BAI) in surviving pine trees, we observed strong positive relationships between the BAI and several individual monoterpenes and diterpenes. Notably, tree age had a negative relationship with the monoterpenes. Furthermore, the outbreak enhanced the resin duct characteristics in lodgepole pine. Our analyses, however, revealed no relationships between the anatomical defence traits, BAI, tree age, size, and NSCs, nor did we find evidence supporting a trade-off between the monoterpenes and diterpenes. These findings offer valuable insights into our broader understanding of conifer defences, particularly concerning how anatomical defences interact in response to outbreaking bark beetle species.

Our study showed strong correlations between monoterpenes and resin duct characteristics. Notably, the overall monoterpenes highly correlated with at least one of the five resin duct characteristics. For instance, the total monoterpenes were linked to the resin duct production, the resin duct size, and the total resin duct area. Furthermore, those monoterpenes that are crucial during MPB host colonization, such as (−)-α-pinene, β-phellandrene, (+)-limonene, and myrcene, had positive relationships with all resin duct characteristics, agreeing with our prior research findings [7,21,51]. Likewise, Kichas et al. [14] reported strong correlations between the resin duct area, resin duct production, and monoterpene concentrations in whitebark pine (*P. albicaulis*). In contrast, we found fewer relationships between the diterpenes and resin duct characteristics, suggesting a weaker association between them. Similarly, Mason et al. [22] reported that the anatomical defence traits of lodgepole pine differentially influenced the different classes of terpenes. Collectively, these findings highlight that the relationship between chemical and anatomical defences may depend on the classes of defence compounds [14]. This is in line with the conclusion from a meta-analysis, suggesting that trade-offs between monoterpenes and resin ducts are relatively rare across a wide range of plant species despite occasional occurrences in some systems [52]. Nonetheless, these results highlight the complementary role of anatomical and chemical defence structures in post-outbreak lodgepole pine stands, likely in response to the MPB outbreaks [7,8,20,22,39,41,42,53].

The second contribution of our study lies in the evidence regarding the positive relationship between total NSCs and total monoterpenes or total diterpenes within the 2015 outbreak. Although such relationships were not consistent among the sites, this result suggests that the production of monoterpenes or diterpenes is linked to NSCs, aligning with previous research findings [29,30,33,54]. For instance, Mullin et al. [55] reported a positive relationship between the starch concentration and total monoterpenes and diterpenes in lodgepole pine trees across varying elevations. Generally, it is well-documented that terpenoid defences require substantial energy demands [32,56,57,58] and deplete carbohydrate reserves [29,30,32]. However, the absence of a similar relationship between anatomical defences and NSCs suggests that our current understanding of the connections between sinks (defences) and sources (NSCs) in conifers is limited and necessitates further field testing [30,37].

One of the strongest relationships we observed is that both the monoterpenes and diterpenes were positively correlated to the tree radial growth, as measured by the BAI. In fact, the BAI exhibited positive relationships with up to 65% of the individual monoterpenes, including (+)-limonene, (−)-α-pinene, myrcene, and β-phellandrene. Similarly, we observed a positive relationship between the individual diterpenes, including dehydroabietic, levopimaric, palustric, neoabietic, and abietic acids, and the BAI in the 2015 outbreak year. It is worth noting that the varying diterpene–BAI relationships between the outbreaks in 2014 and 2015 could be attributed to differences in pine density, as the pine density in the former was nearly double that of the latter.

We observed negative correlations between tree age and the monoterpenes in the 2014 and 2015 outbreak years. Specifically, the age of trees was linked to approximately 45% of the individual monoterpenes in the 2014 outbreak and 40% in the 2015 outbreak. Notably, (+)-limonene, (−)-α-pinene, γ-terpinene, and linalool exhibited the strongest negative associations with tree age. These findings suggest that irrespective of the tree size, tree age may be an important factor in determining the allocation of carbon resources to defence mechanisms. It appears that as trees mature, the allocation of resources to defences becomes less of a priority, whereas younger trees may invest more heavily in defence mechanisms. This could be attributed to the fact that younger trees are potentially more vulnerable to herbivory and thus prioritize defence allocation more than older trees, as supported by Swihart and Bryant [59]. In the case of lodgepole pine, it was found that young trees in the range of 30–50 years old exhibited a greater resistance to ophiostomoid fungi carried by MPBs compared to both younger and older trees [60]. Overall, the relationship between the ontogeny of investment in defence versus growth in long-lived trees remains a complex and unresolved topic within this field of research [2,61].

The MPB outbreak influenced various physiological and growth traits of surviving lodgepole pine trees. Firstly, the post-outbreak conditions improved the resin duct characteristics compared to the pre-outbreak conditions, with the resin duct size, relative resin duct area, and total resin duct area all increasing. This suggests that an increased investment in anatomical defences following MPB outbreaks, agreeing with the results of earlier studies. For instance, Zhao and Erbilgin [8] reported an increased resin duct investment in surviving lodgepole pine trees. Resins serve as the initial line of defence for trees against bark beetle attacks by providing physical and chemical protection [18,25,62,63]. The number and size of xylem resin ducts in pine trees can predict resin production in response to wounding or bark beetle attacks [36,37,39]. Pines that survive MPB attacks tend to have more and larger xylem resin ducts [8,41,42,53] and an increased pre-attack resin production [64]. Based on the anatomical defence and terpene duct characteristics observed in this and other studies, it would be expected that beetles would show a preference for larger pine trees.

Finally, our study did not provide evidence of growth release (BAI) in the surviving lodgepole pine trees. Furthermore, we found no connections between anatomical defence traits and BAI or evidence suggesting any notable relationships between monoterpenes and diterpenes. These findings contradict those of Kichas et al. [14], who reported significant correlations between BAI and resin duct characteristics in whitebark pine and lodgepole pine. We suspect that the enhanced anatomical defences observed after outbreaks may have required additional resources to support their development and maintenance rather than growth. Alternatively, other conifer species, such as spruce, and understory woody plant species, like aspen, may have taken advantage of the available resources following the MPB outbreaks in this study, affecting the growth of surviving lodgepole pine trees. This suggests a complex interplay of factors influencing post-outbreak stand dynamics and resource allocation in these forest ecosystems, highlighting the need for further research to understand these intricate relationships fully.

In conclusion, our retrospective analysis of lodgepole pine trees surviving bark beetle outbreaks revealed a complex interplay between chemical and anatomical defences. Strong correlations between the post-outbreak monoterpene concentrations and resin duct characteristics underscore the importance of these chemical defences in tree resilience, aligning with previous studies on conifer defences [7,14,21]. Although we observed positive relationships between the basal area increment (BAI) and certain monoterpenes and diterpenes, the absence of a significant growth release and the negative correlation between tree age and monoterpenes suggest balanced resource allocation strategies in response to beetle outbreaks. Despite the increased investment in resin ducts and a positive link between NSCs and terpene concentrations, no direct connections were found between anatomical defences and BAIs. These findings indicate that while anatomical and chemical defences are crucial, their interactions and impacts on growth remain complex and warrant further investigations to fully understand the dynamics within post-outbreak forest ecosystems [8,22,29,55,59,60].

## Figures and Tables

**Table 1:** Results of linear mixed-effect models showing the relationship between total monoterpenes and each of the five annual resin duct characteristics of lodgepole pine trees in Jasper National Park (Alberta, Canada) for different mountain pine beetle outbreak years and periods.

Outbreak Years	Period	Variables	Total Monoterpenes		
			Coefficient	*t*-Value	*p*-Value
2014	Post-outbreak	Resin duct production	1.1 × 10^3^	4.3	***<0.001***
		Total resin duct area	4.8 × 10^6^	4.8	***<0.001***
		Individual resin duct size	4.2 × 10^7^	2.4	***0.029***
		Resin duct density	1.4 × 10^3^	0.5	0.626
		Relative resin duct area (%)	2.2 × 10^5^	1.4	0.187
	5 years pre-outbreak	Resin duct production	1.1 × 10^3^	3.1	***0.005***
		Total resin duct area	4.8 × 10^6^	3.2	***0.005***
		Individual resin duct size	3.0 × 10^7^	1.7	0.107
		Resin duct density	2.7 × 10^3^	0.5	0.637
		Relative resin duct area (%)	4.2 × 10^5^	1.4	0.189
	10 years pre-outbreak	Resin duct production	1.4 × 10^3^	4.2	***<0.001***
		Total resin duct area	6.2 × 10^6^	4.1	***<0.001***
		Individual resin duct size	6.0 × 10^7^	2.4	***0.025***
		Resin duct density	1.5 × 10^3^	0.3	0.761
		Relative resin duct area (%)	4.2 × 10^5^	1.3	0.219
2015	Post-outbreak	Resin duct production	78.7	0.2	0.831
		Total resin duct area	3.4 × 10^6^	2.1	***0.045***
		Individual resin duct size	2.3 × 10^7^	4.2	***<0.001***
		Resin duct density	−100.8	−0.3	0.767
		Relative resin duct area (%)	−1.3 × 10^3^	−0.1	0.946
	5 years pre-outbreak	Resin duct production	584.0	1.6	0.127
		Total resin duct area	5.8 × 10^6^	3.3	***0.002***
		Individual resin duct size	3.0 × 10^7^	3.7	***0.002***
		Resin duct density	−127.4	−0.2	0.830
		Relative resin duct area (%)	−8.0 × 10^3^	−0.2	0.868
	10 years pre-outbreak	Resin duct production	741.6	1.8	0.081
		Total resin duct area	6.2 × 10^6^	3.3	***0.002***
		Individual resin duct size	4.0 × 10^7^	4.1	***<0.001***
		Resin duct density	−280.3	−0.3	0.762
		Relative resin duct area (%)	−6.8 × 10^3^	−0.1	0.926

Significant *p*-values (α = 0.05) are bolded and italicized.

**Table 2:** Results of linear mixed-effect models showing the direction of relationships between individual monoterpenes and the selected anatomical defence characteristics of lodgepole pine in Jasper National Park (Alberta, Canada) for different mountain pine beetle outbreak years and periods.

Monoterpenes	2014 Outbreak								2015 Outbreak					
	Post-Outbreak			5 Years Pre-Outbreak		10 Years Pre-Outbreak			Post-Outbreak		5 Years Pre-Outbreak		10 Years Pre-Outbreak	
	RDP	RDA	RDS	RDP	RDA	RDP	RDA	RDS	RDA	RDS	RDA	RDS	RDA	RDS
4-allylanisole														
(−)-borneol	++	++		++	++	+	+	+	+	++	+	+	+	++
Bornyl acetate		+	+	+	+		+	+						
(+)-camphene	++	++		++	++	++	++	+		+++	+	+++		+++
3-carene								+				+		+
(+)-limonene	+++	+++	+	++	++	+++	+++		+	+++	++	++	++	+++
(−)-limonene														
Linalool		+	++	++	+++	+++	++	++	+	++	+	++	++	++
Myrcene	++	+++		++	++	+++	+++		+	+++	++	+++	++	++
Ocimene	++	++		+++	+++	+++	+++							
β-phellandrene	++	++	+	+	++	+++	+++		+	+++	+	++	+	+++
(+)-α-pinene	+	++		+	+		+							
(−)-α-pinene	+++	+++	+	+++	+++	+++	+++	+	+	+++	+++	++	+++	+++
(+)-β-pinene	+	++	+	+	+	+	+	++	+	+	+	+	+	++
(−)-β-pinene	++	+++		++	+	++	++		++	+	++		+++	
β-phellandrene	++	++	+	+	++	+++	+++		+	+++	+	++	+	+++
(R)-(+)-pulegone		+	+	++	++	+	++	++						
α-terpinene									+		+		+	
γ-terpinene	+	+	+					++	+	+	++	++	+	++
α-terpineol										+++		++	+	+++
Terpinolene									++		+++	+	+	+

RDP, resin duct production; RDA, total resin duct area; RDS, individual resin duct size. + denotes a positive relationship, and − denotes a negative relationship. +, *p*-value ≤ 0.05; ++, *p*-value ≤ 0.01; +++, *p*-value ≤ 0.001.

**Table 3:** Results of linear mixed-effect models showing the relationship between total monoterpenes and total diterpenes and the annual basal area increment (BAI) of lodgepole pine trees in Jasper National Park (Alberta, Canada) for different mountain pine beetle outbreak years and periods.

Outbreak Years	BAI	Total Monoterpenes			Total Diterpenes		
		Coefficient	*t*-Value	*p*-Value	Coefficient	*t*-Value	*p*-Value
2014	Post-outbreak	532.4	2.7	***0.015***	−0.8	−0.5	0.608
	5 years pre-outbreak	549.9	2.6	***0.015***	−0.8	−0.5	0.658
	10 years pre-outbreak	665.1	3.2	***0.004***	−1.2	−0.7	0.516
2015	Post-outbreak	485.9	2.6	***0.013***	4.9	2.7	***0.012***
	5 years pre-outbreak	686.8	3.7	***0.002***	4.9	2.4	***0.024***
	10 years pre-outbreak	791.7	3.7	***0.001***	6.3	2.6	***0.013***

Significant *p*-values (α = 0.05) are bolded and italicized.

**Table 4:** Results of linear mixed-effect models showing the direction of relationships between individual monoterpenes or diterpenes and the annual basal area increment (BAI) of lodgepole pine in Jasper National Park (Alberta, Canada) for different mountain pine beetle outbreak years and periods. Since none of the results for diterpenes are statistically different in the 2014 outbreak year, only the results from the 2015 outbreak year are presented.

Terpene Classes	2014 Outbreak			2015 Outbreak		
	Post-Outbreak BAI	5-Year Pre-Outbreak BAI	10-Year Pre-Outbreak BAI	Post-Outbreak BAI	5-Year Pre-Outbreak BAI	10-Year Pre-Outbreak BAI
Monoterpenes						
4-allylanisole						
(−)-borneol	+++	+	++			
Bornyl acetate	++	+	+			
(+)-camphene	++	++	++	+	+	+
3-carene						
(+)-limonene	+	+	++	++	+++	+++
(−)-Limonene						
linalool	+++	+++	+++	+++	++	+++
Myrcene	++	++	+++	++	+++	+++
Ocimene	+	+	++			
β-phellandrene	+	++	++	++	++	+++
(+)-α-pinene	++		+			
(−)-α-pinene	+++	++	+++	++	+++	+++
(+)-β-pinene	+	+	+	+	+	+
(−)-β-pinene	++	+	++		++	++
(R)-(+)-Pulegone	++	+++	+++			
α-terpinene						
γ-terpinene				+	++	++
α-terpineol			+	+++	+++	+++
Terpinolene				+	+	+
Diterpenes						
Abietic				+	+	+
Dehydroabietic				++	++	+++
Levopiramic				+++	+++	+++
Neoabietic				+++	+++	+++
Palustric				++	++	+++
Sandaracopiramic						

+ denotes a positive relationship, and − denotes a negative relationship. +, *p*-value ≤ 0.05; ++, *p*-value ≤ 0.01; +++, *p*-value ≤ 0.001.

**Table 5:** Results of linear mixed-effect models showing the direction of relationships between individual monoterpenes and age of lodgepole pine trees in Jasper National Park (Alberta, Canada) for different mountain pine beetle outbreak years.

	Tree Age	
Monoterpenes	2014 Outbreak	2015 Outbreak
4-allylanisole		
(−)-borneol		
Bornyl acetate		
(+)-camphene	-	
3-carene		
(+)-limonene	-	--
(−)-limonene		
Linalool	---	--
Myrcene		-
Ocimene		
β-phellandrene		-
(+)-α-pinene		
(−)-α-pinene	-	--
(+)-β-pinene	-	
(−)-β-pinene		-
(R)-(+)-pulegone	-	
α-terpinene	-	
γ-terpinene	--	--
α-terpineol	--	
Terpinolene		-

+ denotes a positive relationship, and − denotes a negative relationship. -, *p*-value ≤ 0.05; --, *p*-value ≤ 0.01; ---, *p*-value ≤ 0.001.

## Data Availability

The original contributions presented in the study are included in the article/Supplementary Material; further inquiries can be directed to the corresponding author.

## Supplementary Materials

The following supporting information can be downloaded at: https://www.mdpi.com/article/10.3390/metabo14090472/s1, Table S1. Total and live and dead lodgepole pine density (trees/ha) at five study sites. Total and pine densities were calculated using basal area factor 4.6 metric (20 standards) from variable radius plots and the diameter at the breast height of trees. Sites were categorized based on the year of the mountain pine beetle outbreak at each site. Table S2. Concentrations (mean ± SE) of monoterpenes, diterpenes and non-structural carbohydrates of live lodgepole pine among five sites located in the Jasper National Park (Alberta, Canada) in the 2014 and 2015 outbreak years. Table S3. Results of indirect gradient analysis by NMDS with Bray–Curtis dissimilarity showing the relationship between non-structural carbohydrates and monoterpenes or diterpenes of lodgepole pine in Jasper National Park (Alberta, Canada) in the 2014 and 2015 outbreak years. Table S4. Results of indirect gradient analysis by NMDS with Bray–Curtis dissimilarity showing the relationship between the annual basal area increment (BAI, mm^2^ year^−1^) and diterpenes or non-structural carbohydrates of lodgepole pine trees in Jasper National Park (Alberta, Canada) in the 2014 and 2015 outbreak years. Table S5. Results of indirect gradient analysis by NMDS with Bray–Curtis dissimilarity results showing the relationship of total monoterpenes with diameter at breast height (DBH) and age of lodgepole pine trees in Jasper National Park (Alberta, Canada) in the 2014 and 2015 outbreak years. Table S6. Results of indirect gradient analysis by NMDS with Bray–Curtis dissimilarity showing the relationship of non-structural carbohydrates and each of the five annual resin duct characteristics of lodgepole pine trees in Jasper National Park (Alberta, Canada) in the 2014 and 2015 outbreak years. Table S7. Results of indirect gradient analysis by NMDS with Bray–Curtis dissimilarity showing the relationship between monoterpenes and individual diterpenes of lodgepole pine in Jasper National Park (Alberta, Canada) in the 2014 and 2015 outbreak years. Figure S1. Results of indirect gradient analysis by NMDS with Bray–Curtis dissimilarity showing the relationship between monoterpenes and non-structural carbohydrates of lodgepole pine trees in Jasper National Park (Alberta, Canada) in the 2014 and 2015 outbreak years. Figure S2. Results of indirect gradient analysis by NMDS with Bray–Curtis dissimilarity showing the relationship between diterpenes and non-structural carbohydrates of lodgepole pine trees in Jasper National Park (Alberta, Canada) in the 2014 and 2015 outbreak years. Figure S3. Results of indirect gradient analysis by NMDS with Bray–Curtis dissimilarity showing the relationship between individual diterpenes and diameter at breast height (DBH) and age of lodgepole pine trees in Jasper National Park (Alberta, Canada) in the 2014 and 2015 outbreak years. Figure S4. Results of indirect gradient analysis by NMDS with Bray–Curtis dissimilarity showing the relationship between non-structural carbohydrates and each of the five annual resin duct characteristics over post-outbreak (2014A, 2015A) and 5-year and 10-year pre-outbreak (2014B, 2015B) of lodgepole pine trees in Jasper National Park (Alberta, Canada). The outbreaks occurred in 2014 or 2015. RDP: Resin duct production; RDA: Total resin duct area; RDS: Individual resin duct size; RDD: Resin duct density; RDA%: Relative resin duct area; 5yB: 5-year pre-outbreak; 10yB: 10-year pre-outbreak. Figure S5. Results of indirect gradient analysis by NMDS with Bray–Curtis dissimilarity showing the relationship between non-structural carbohydrates and the annual basal area increment (BAI, mm^2^ year^−1^) over post-outbreak (2014A, 2015A) and 5-year and 10-year pre-outbreak (2014B, 2015B) of lodgepole pine trees in Jasper National Park (Alberta, Canada) in the 2014 and 2015 outbreak years. 5yB: 5-year pre-outbreak; 10yB: 10-year pre-outbreak. Figure S6. Results of indirect gradient analysis by NMDS with Bray–Curtis dissimilarity showing the relationship between non-structural carbohydrates and diameter at breast height (DBH) and age of lodgepole pine trees in Jasper National Park (Alberta, Canada) in the 2014 and 2015 outbreak years. Figure S7. Results of indirect gradient analysis by NMDS with Bray–Curtis dissimilarity showing the relationship between monoterpenes and diterpenes of lodgepole pine trees in Jasper National Park (Alberta, Canada) in the 2014 and 2015 outbreak years.

## Abbreviations

BAI	Basal area increment
DBH	Diameter at breast height
DW	Dry weight
ELSD	Evaporative light scattering detector
GC/MS	Gas chromatograph/mass spectrometer
MPB	Mountain pine beetle
NMDS	Non-linear multidimensional scaling
NSCs	Non-structural carbohydrates
UHPLC	Ultra-high performance liquid chromatograph

## References

[1] Raffa K.F., Aukema B.H., Bentz B.J., Carroll A.L., Hicke J.A., Turner M.G., Romme W.H. (2008). Cross-scale drivers of natural disturbances prone to anthropogenic amplification: The dynamics of bark beetle eruptions. BioScience.

[2] Bentz B.J., Régnière J., Fettig C.J., Hansen E.M., Hayes J.L., Hicke J.A., Kelsey R.G., Negrón J.F., Seybold S.J. (2010). Climate change and bark beetles of the western United States and Canada: Direct and indirect effects. BioScience.

[3] Kausrud K., Økland B., Skarpaas O., Grégoire J., Erbilgin N., Stenseth N.C. (2011). Population dynamics in changing environments: The case of an eruptive forest pest species. Biol Rev..

[4] Seidl R., Schelhaas M.-J., Rammer W., Verkerk P.J. (2014). Increasing forest disturbances in Europe and their impact on carbon storage. Nat. Clim. Chang..

[5] Ghimire B., Williams C.A., Collatz G.J., Vanderhoof M., Rogan J., Kulakowski D., Masek J.G. (2015). Large carbon release legacy from bark beetle outbreaks across Western United States. Glob. Chang. Biol..

[6] Aldea J., Dahlgren J., Holmström E., Löf M. (2023). Current and future drought vulnerability for three dominant boreal tree species. Glob. Chang. Biol..

[7] Erbilgin N., Cale J.A., Hussain A., Ishangulyyeva G., Klutsch J.G., Najar A., Zhao S. (2017). Weathering the storm: How lodgepole pine trees survive mountain pine beetle outbreaks. Oecologia.

[8] Zhao S., Erbilgin N. (2019). Larger resin ducts are linked to the survival of lodgepole pine trees during mountain pine beetle outbreak. Front Plant Sci..

[9] Amoroso M.M., Coates K.D., Astrup R. (2013). Stand recovery and self-organization following large-scale mountain pine beetle induced canopy mortality in northern forests. For. Ecol. Manag..

[10] Perovich C., Sibold J.S. (2016). Forest composition change after a mountain pine beetle outbreak, Rocky Mountain National Park, CO, USA. For. Ecol. Manag..

[11] Morris J.E., Buonanduci M.S., Agne M.C., Battaglia M.A., Harvey B.J. (2021). Does the legacy of historical thinning treatments foster resilience to bark beetle outbreaks in subalpine forests?. Ecol Appl..

[12] Rodman K.C., Andrus R.A., Carlson A.R., Carter T.A., Chapman T.B., Coop J.D., Fornwalt P.J., Gill N.S., Harvey B.J., Hoffman A.E. (2022). Rocky Mountain forests are poised to recover following bark beetle outbreaks but with altered composition. J. Ecol..

[13] Lieffers V.J., Benedik J., Stadt K., Macdonald S.E. (2024). Poor regeneration of pine after mountain pine beetle attack in colder boreal regions of Canada. Can. J. For. Res..

[14] Kichas N.E., Trowbridge A.M., Raffa K.F., Malone S.C., Hood S.M., Everett R.G., McWethy D.B., Pederson G.T. (2021). Growth and defense characteristics of whitebark pine (*Pinus albicaulis*) and lodgepole pine (*Pinus contorta* var. *latifolia*) in a high-elevation, disturbance-prone mixed-conifer forest in northwestern Montana, USA. For. Ecol. Manag..

[15] Marini L., Økland B., Jönsson A.M., Bentz B., Carroll A., Forster B., Grégoire J., Hurling R., Nageleisen L.M., Netherer S. (2017). Climate drivers of bark beetle outbreak dynamics in Norway spruce forests. Ecography.

[16] Thom D., Rammer W., Seidl R. (2017). The impact of future forest dynamics on climate: Interactive effects of changing vegetation and disturbance regimes. Ecol. Monogr..

[17] Sommerfeld A., Rammer W., Heurich M., Hilmers T., Müller J., Seidl R. (2020). Do bark beetle outbreaks amplify or dampen future bark beetle disturbances in Central Europe?. J. Ecol..

[18] Franceschi V.R., Krokene P., Christiansen E., Krekling T. (2005). Anatomical and chemical defenses of conifer bark against bark beetles and other pests. New Phytol..

[19] Xu D., Xu L., Zhou F., Wang B., Wang S., Lu M., Sun J. (2018). Gut bacterial communities of *Dendroctonus valens* and monoterpenes and carbohydrates of *Pinus tabuliformis* at different attack densities to host pines. Front. Microbiol..

[20] Vázquez-González C., Zas R., Erbilgin N., Ferrenberg S., Rozas V., Sampedro L. (2020). Resin ducts as resistance traits in conifers: Linking dendrochronology and resin-based defences. Tree Physiol..

[21] Erbilgin N. (2018). Phytochemicals as mediators for host range expansion of a native invasive forest insect herbivore. New Phytol..

[22] Mason C.J., Keefover-Ring K., Villari C., Klutsch J.G., Cook S., Bonello P., Erbilgin N., Raffa K.F., Townswend P.A. (2019). Anatomical defenses against bark beetles related to degree of historical exposure between species and are allocated independently of chemical defenses within trees. Plant Cell Environ..

[23] Nagel R., Hammerbacher A., Kunert G., Phillips M.A., Gershenzon J., Schmidt A. (2022). Bark beetle attack history does not influence the induction of terpene and phenolic defenses in mature Norway spruce (*Picea abies*) trees by the bark beetle-associated fungus Endoconidiophora polonica. Front Plant Sci..

[24] Mageroy M.H., Nagy N.E., Steffenrem A., Krokene P., Hietala A.M. (2023). Conifer Defences against Pathogens and Pests Mechanisms, Breeding, and Management. Curr. For. Rep..

[25] Chiu C.C., Keeling C.I., Bohlmann J. (2017). Toxicity of pine monoterpenes to mountain pine beetle. Sci. Rep..

[26] Ullah A., Klutsch J.G., Erbilgin N. (2021). Production of complementary defense metabolites reflects a co-evolutionary arms race between a host plant and a mutualistic bark beetle-fungal complex. Plant Cell Environ..

[27] Chiu C.C., Bohlmann J. (2022). Mountain pine beetle epidemic: An interplay of terpenoids in host defense and insect pheromones. Annu. Rev. Plant Biol..

[28] Schiebe C., Hammerbacher A., Birgersson G., Witzell J., Brodelius P.E., Gershenzon J., Hansson B.S., Krokene P., Schlyter F. (2012). Inducibility of chemical defenses in Norway spruce bark is correlated with unsuccessful mass attacks by the spruce bark beetle. Oecologia.

[29] Goodsman D.W., Lusebrink I., Landhäusser S.M., Erbilgin N., Lieffers V.J. (2012). Variation in carbon availability, defense chemistry and susceptibility to fungal invasion along the stems of mature trees. New Phytol..

[30] Erbilgin N., Zanganeh L., Klutsch J.G., Chen S., Zhao S., Ishangulyyeva G., Burr S.J., Gaylord M., Hofstetter R., Keefover-Ring K. (2021). Combined drought and bark beetle attacks deplete non-structural carbohydrates and promote death of mature pine trees. Plant, Cell Environ..

[31] Hartmann H., Trumbore S. (2016). Understanding the roles of non-structural carbohydrates in forest trees—From what we can measure to what we want to know. New Phytol..

[32] Wiley E., Rogers B.J., Hodgkinson R., Landhäusser S.M. (2015). Non-structural carbohydrate dynamics of lodgepole pine dying from mountain pine beetle attack. New Phytol..

[33] Huang J.B., Kautz M., Trowbridge A.M., Hammerbacher A., Raffa K.F., Adams H.D., Goodsman D.W., Xu C., Meddens A.J.H., Kandasamy D. (2020). Tree defense and bark beetles in a drying world: Carbon partitioning, functioning and modeling. New Phytol..

[34] Lahr E.C., Krokene P. (2013). Conifer stored resources and resistance to a fungus associated with the spruce bark beetle *Ips typographus*. PLoS ONE.

[35] Zas R., Moreira X., Ramos M., Lima M.R.M., Da Silva M.N., Solla A., Vasconcelos M.W., Sampedro L. (2014). Intraspecific variation of anatomical and chemical defensive traits in Maritime pine (*Pinus pinaster*) as factors in susceptibility to the pinewood nematode (*Bursaphelenchus xylophilus*). Trees—Struct Func..

[36] Blanche C.A., Lorio P.L., Sommers R.A., Hodges J.D., Nebeker T.E. (1992). Seasonal cambial growth and development of loblolly pine: Xylem formation, inner bark chemistry, resin ducts, and resin flow. For. Ecol. Manag..

[37] Lombardero M.J., Ayres M.P., Lorio P.L., Ruel J.J. (2000). Environmental effects on constitutive and inducible resin defences of Pinus taeda. Ecol. Lett..

[38] Rodríguez-García A., López R., Martín J.A., Pinillos F., Gil L. (2014). Resin yield in *Pinus pinaster* is related to tree dendrometry, stand density and tapping-induced systemic changes in xylem anatomy. For. Ecol. Manag..

[39] Hood S., Sala A. (2015). Ponderosa pine resin defenses and growth: Metrics matter. Tree Physiol..

[40] Westbrook J.W., Walker A.R., Neves L.G., Munoz P., Resende M.F., Neale D.B., Wegrzyn J.L., Huber D.A., Kirst M., Davis J.M. (2015). Discovering candidate genes that regulate resin canal number in *Pinus taeda* stems by integrating genetic analysis across environments, ages, and populations. New Phytol..

[41] Kane J.M., Kolb T.E. (2010). Importance of resin ducts in reducing ponderosa pine mortality from bark beetle attack. Oecologia.

[42] Ferrenberg S., Kane J.M., Mitton J.B. (2013). Resin duct characteristics associated with tree resistance to bark beetles across lodgepole and limber pines. Oecologia.

[43] Hood S., Sala A., Heyerdahl E.K., Boutin M. (2015). Low-severity fire increases tree defense against bark beetle attacks. Ecology.

[44] Cale J.A., Klutsch J.G., Dykstra C.B., Peters B., Erbilgin N. (2019). Pathophysiological responses of pine defensive metabolites largely lack differences between pine species but vary with eliciting ophiostomatoid fungal species. Tree Physiol..

[45] Kersten P.J., Kopper B.J., Raffa K.F., Illman B.L. (2006). Rapid analysis of abietanes in conifers. J. Chem. Ecol..

[46] R Core Team 2018 R: A Language and Environment for Statistical Computing R Foundation for Statistical Computing. Vienna, Austria. https://wwwR-projectorg/.

[47] Harrell F.E. (2021). Hmisc: Harrell Miscellaneous. R Package Version 4.6-0. https://CRAN.R-project.org/package=Hmisc.

[48] Oksanen J., Simpson G.L., Blanchet F.G., Kindt R., Legendre P., Minchin P.R., O’Hara R.B., Solymos P., Stevens M.H.H., Szoecs E. (2020). Vegan: Community Ecology Package. R Package Version 2.5-7. https://CRAN.R-project.org/package=vegan.

[49] Wickham H. (2016). Ggplot2: Elegant Graphics for Data Analysis.

[50] Bates D., Mächler M., Bolker B., Walker S. (2014). Fitting linear mixed-effects models using lme4. J. Stat. Soft..

[51] Erbilgin N., Ma C., Whitehouse C., Shan B., Najar A., Evenden M. (2014). Chemical similarity between historical and novel host plants promotes range and host expansion of the mountain pine beetle in a naïve host ecosystem. New Phytol..

[52] Moles A.T., Peco B., Wallis I.R., Foley W.J., Poore A.G.B., Seabloom E.W., Vesk P.A., Bisigato A.J., Cella-Pizarro L., Clark C.J. (2013). Correlations between physical and chemical defences in plants: Tradeoffs, syndromes, or just many different ways to skin a herbivorous cat?. New Phytol..

[53] Gaylord M.L., Kolb T.E., McDowell N.G. (2015). Mechanisms of pion pine mortality after severe drought: A retrospective study of mature trees. Tree Physiol..

[54] Dietze M.C., Sala A., Carbone M.S., Czimczik C.I., Mantooth J.A., Richardson A.D., Vargas R. (2014). Non-structural carbon in woody plans. Ann Rev Plant Biol..

[55] Mullin M., Klutsch J.G., Cale J.A., Hussain A., Zhao S., Whitehouse C., Erbilgin N. (2021). Primary and secondary metabolite profiles of lodgepole pine trees change with elevation, but not with latitude. J. Chem. Ecol..

[56] Herms D.A., Mattson W.J. (1992). The dilemma of plants: To grow or defend. Q. Rev. Biol..

[57] Gershenzon J. (1994). Metabolic costs of terpenoid accumulation in higher plants. J. Chem. Ecol..

[58] Kolb T., Keefover-Ring K., Burr S.J., Hofstetter R., Gaylord M., Raffa K.F. (2019). Drought-mediated changes in tree physiological processes weaken tree defenses to bark beetle attack. J. Chem. Ecol..

[59] Swihart R.K., Bryant J.P. (2001). Importance of biogeography and ontogeny of woody plants in winter herbivory by mammals. J. Mammal..

[60] Shrimpton D.M. (1973). Age-and size-related response of lodgepole pine to inoculation with *Europhium clavigerum*. Can. J. Bot..

[61] Hartmann H., Moura C.F., Anderegg W.R.L., Ruehr N.K., Salmon Y., Allen C.D., Arndt S.K., Breshears D.D., Davi H., Galbraith D. (2018). Research frontiers for improving our understanding of drought-induced tree and forest mortality. New Phytol..

[62] Seybold S.J., Huber D.P.W., Lee J.C., Graves A.D., Bohlmann J. (2006). Pine monoterpenes and pine bark beetles: A marriage of convenience for defense and chemical communication. Phytochem. Rev..

[63] Reid M.L., Purcell J.R.C. (2011). Condition-dependent tolerance of monoterpenes in an insect herbivore. Arthropod-Plant Interact..

[64] Shrimpton D.M., Reid R.W. (1973). Change in resistance of lodgepole pine to mountain pine beetle between 1965 and 1972. Can. J. For. Res..

