# Supplementary material for: The Legacy Effect of Mountain Pine Beetle Outbreaks on the Chemical and Anatomical Defences of Surviving Lodgepole Pine Trees
Source: Metabolites. 2024 Aug 27;14(9):472. doi: 10.3390/metabo14090472 (PMC11434468; doi:10.3390/metabo14090472)
Supplement: Supplementary file 1 [file metabolites-14-00472-s001.zip › metabolites-3166683-supplementary.pdf]

Table S1. Total and live and dead lodgepole pine density (trees/ha) at five study sites. Total and pine densities were calculated using basal area factor 4.6 metric (20 standards) from variable radius plots and the diameter at the breast height of trees. Sites were categorized based on the year of the mountain pine beetle outbreak at each site.

Outbreak Years	Site	Total (trees ha^{-1})	Live pine (trees ha^{-1})	Dead pine (trees ha^{-1})
2015	1	1150.88	506.18	173.89
	2	685.39	310.48	57.45
	3	523.13	286.78	236.35
2014	4	1283.11	824.50	485.60
	5	904.88	0	904.88

Table S2. Concentrations (mean ± SE) of monoterpenes, diterpenes and non-structural carbohydrates of live lodgepole pine among five sites located in the Jasper National Park (Alberta, Canada) in the 2014 and 2015 outbreak years.

Chemicals	Site 1-3 (2015)	Sites 4-5 (2014)
	**Monoterpenes**	
4-Allylanisole	84.17±16.6	111.38±18.9
(-)-Borneol	4.27±1.0	12.65±4.2
Bornyl acetate	19.86±8.4	51.30±19.8
(+)-Camphene	38.40±7.2	157.87±28.7
3-Carene	404.05±70.9	372.87±128.0
(+)-Limonene	42.27±4.1	67.18±7.2
(-)-Limonene	566.02±190.2	635.48±214.1
Linalool	0.93±0.3	2.31±0.5
Myrcene	105.39±10.3	178.00±22.9
Ocimine	34.27±8.3	206.28±52.0
β-Phellandrene	3,563.50±377.6	5,205.14±590.56
(+)-α-Pinene	147.11±44.3	232.69±90.6
(-)-α-Pinene	90.47±9.8	182.09±26.8
(+)-β-Pinene	28.42±3.8	45.18±8.6
(-)-β-Pinene	397.65±61.4	1149.71±211.9
R-(+)-Pulegone	1.27±0.4	1.07±0.4
α-Terpinene	64.46±10.3	101.35±17.4
γ-Terpinene	9.11±1.4	14.26±2.5
α-Terpineol	2.21±0.8	9.59±1.8
Terpinolene	85.21±12.5	257.76±68.3
Total	***5,698.01±560.5***	***8,988.17±1028.2***
	**Diterpenes**	
Abietic	16.12±1.4	14.35±2.2
Dehydroabietic	3.51±0.4	3.16±0.4
Levopiramic	12.82±1.4	9.82±1.4
Neoabietic	4.00±0.5	2.53±0.4
Palustric	5.97±0.5	5.17±0.8
Sandaracopiramic	6.81±1.9	4.14±1.3
Total	***48.84±4.4***	***38.19±6.1***
	**Non-structural carbohydrates**	
Fructose	5.64±0.5	6.77±0.6
Glucose	9.82±0.3	10.91±0.3
Starch	71.93±2.3	79.33±2.2
Sucrose	34.82±0.8	31.68±1.1
Total sugars	50.28±1.4	49.36±1.5
Total	***122.21±3.3***	***128.69±3.1***

Table S3. Results of indirect gradient analysis by NMDS with Bray–Curtis dissimilarity showing the relationship between non-structural carbohydrates and monoterpenes or diterpenes of lodgepole pine in Jasper National Park (Alberta, Canada) in the 2014 and 2015 outbreak years.

**Variables**	**2014-Outbreak**		**2015-Outbreak**	
	**r^2**	***p*-value**	**r^2**	***p*-value**
		Monoterpenes		
Fructose	0.180	0.185	0.027	0.656
Glucose	0.066	0.567	0.037	0.586
Sucrose	0.034	0.715	0.096	0.253
Starch	0.024	0.790	0.104	0.202
		Diterpenes		
Fructose	0.064	0.580	0.006	0.902
Glucose	0.129	0.396	0.173	0.065
Sucrose	0.165	0.211	0.022	0.727
Starch	0.055	0.585	0.105	0.194

r^2 represents the proportion of the variance for monoterpenes or diterpenes, which was explained by projecting each non-structural carbohydrate to the NMDS ordination axis. Significant *p*-values (α=0.05) were bolded and italicized.

Table S4. Results of indirect gradient analysis by NMDS with Bray–Curtis dissimilarity showing the relationship between the annual basal area increment (BAI, mm^2 $year^{-1}$) and diterpenes or non-structural carbohydrates of lodgepole pine trees in Jasper National Park (Alberta, Canada) in the 2014 and 2015 outbreak years.

**BAI**	**2014-Outbreak**		**2015-Outbreak**	
	**r^2**	***p*-value**	**r^2**	***p*-value**
	Diterpenes			
Post-outbreak	0.029	0.769	0.115	0.162
5-yr pre-outbreak	0.023	0.850	0.163	0.071
10-yr pre-outbreak	0.019	0.854	0.189	***0.050***
	Non-Structural Carbohydrates			
Post-outbreak	0.059	0.568	0.116	0.175
5-yr pre-outbreak	0.014	0.881	0.083	0.276
10-yr pre-outbreak	0.001	0.990	0.094	0.232

r^2 shows the proportion of the variance for diterpene or non-structural carbohydrates which was explained by projecting BAI to the NMDS ordination axis. Significant *p*-values (α=0.05) were bolded and italicized.

Table S5. Results of indirect gradient analysis by NMDS with Bray–Curtis dissimilarity results showing the relationship of total monoterpenes with diameter at breast height (DBH) and age of lodgepole pine trees in Jasper National Park (Alberta, Canada) in the 2014 and 2015 outbreak years.

**Outbreak Year**	**Variables**	**Total Monoterpenes**	
		**r^2**	***p*-value**
2014	DBH	0.039	0.693
	Age	0.451	***0.001***
2015	DBH	0.024	0.695
	Age	0.243	***0.017***

r^2 shows the proportion of the variance for monoterpenes, diterpenes, and NSCs which was explained by projecting DBH and age to the NMDS ordination axis. Significant *p*-values (α=0.05) were bolded and italicized.

Table S6. Results of indirect gradient analysis by NMDS with Bray–Curtis dissimilarity showing the relationship of non-structural carbohydrates and each of the five annual resin duct characteristics of lodgepole pine trees in Jasper National Park (Alberta, Canada) in the 2014 and 2015 outbreak years.

**Periods**	**Variables***	**2014-Outbreak**		**2015-Outbreak**	
		r^2	***p*-value**	r^2	***p*-value**
Post-outbreak	RDP	0.145	0.231	0.011	0.851
	RDA	0.045	0.667	0.122	0.141
	RDS	0.131	0.294	0.158	0.075
	RDD	0.158	0.205	0.017	0.780
	RDA%	0.043	0.662	0.034	0.590
5-yr pre-outbreak	RDP	0.044	0.699	0.042	0.499
	RDA	0.006	0.936	0.112	0.173
	RDS	0.217	0.129	0.069	0.360
	RDD	0.054	0.589	0.007	0.913
	RDA%	0.015	0.873	0.025	0.667
10-yr pre-outbreak	RDP	0.068	0.544	0.028	0.627
	RDA	0.016	0.851	0.086	0.255
	RDS	0.027	0.789	0.070	0.357
	RDD	0.178	0.156	0.002	0.969
	RDA%	0.080	0.487	0.015	0.794

* RDP: Resin duct production; RDA: Total resin duct area; RDS: Individual resin duct size; RDD: Resin duct density; RDA%: Relative resin duct area. r^2 shows the proportion of the variance for the carbohydrate profile which was explained by projecting resin duct and growth characteristics to the NMDS ordination axis. Significant *p*-values (α=0.05) were bolded and italicized.

Table S7. Results of indirect gradient analysis by NMDS with Bray–Curtis dissimilarity showing the relationship between monoterpenes and individual diterpenes of lodgepole pine in Jasper National Park (Alberta, Canada) in the 2014 and 2015 outbreak years.

Variables	2014-Outbreak		2015-Outbreak	
	r^2	*p*-value	r^2	*p*-value
Abietic	0.056	0.611	0.010	0.866
Dehydroabietic	0.006	0.945	0.051	0.474
Levopiramic	0.051	0.620	0.022	0.715
Neoabietic	0.071	0.525	0.013	0.825
Palustric	0.051	0.626	0.036	0.582
Sandaracopiramic	0.061	0.581	0.026	0.740

r^2 represents the proportion of the variance for monoterpenes which was explained by projecting each diterpene to the NMDS ordination axis. Significant *p*-values (α=0.05) were bolded and italicized.

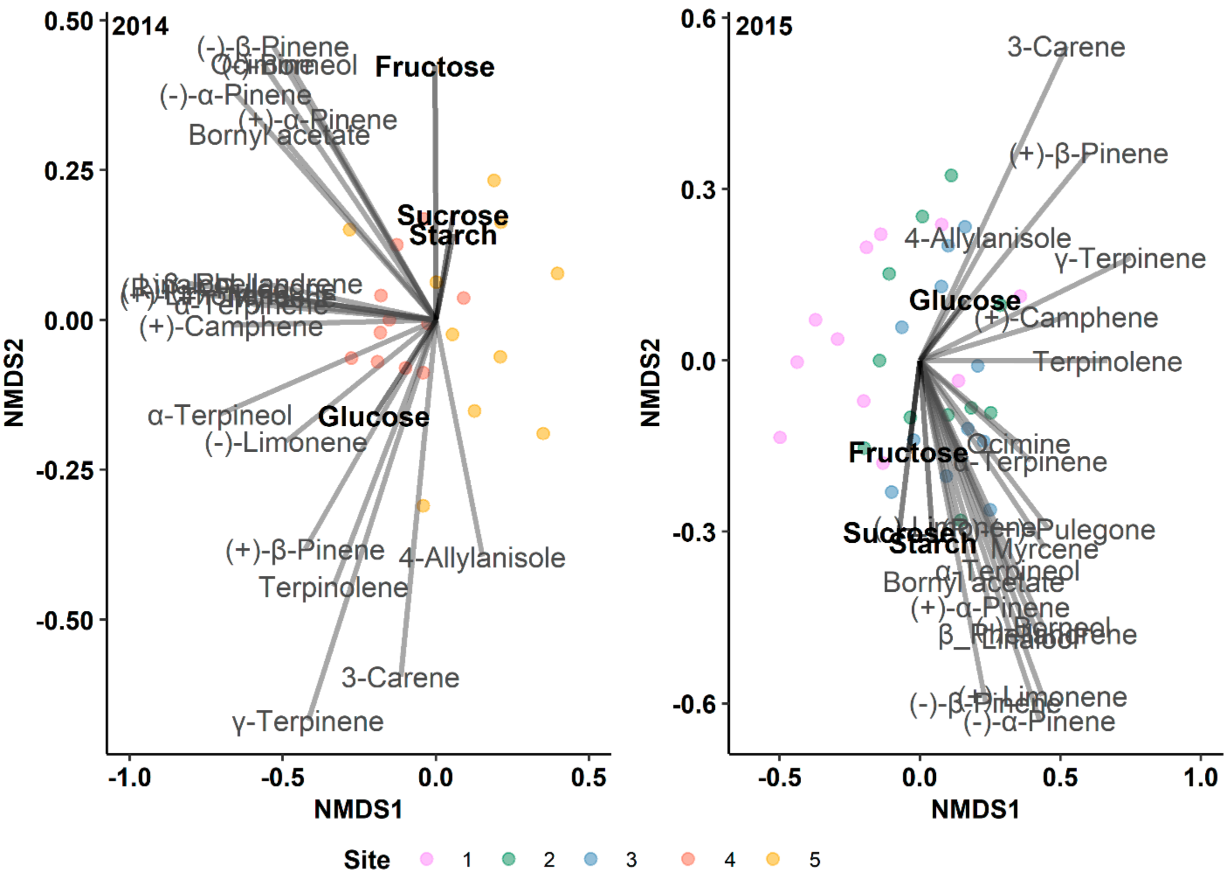

Figure S1. Results of indirect gradient analysis by NMDS with Bray–Curtis dissimilarity showing the relationship between monoterpenes and non-structural carbohydrates of lodgepole pine trees in Jasper National Park (Alberta, Canada) in the 2014 and 2015 outbreak years.

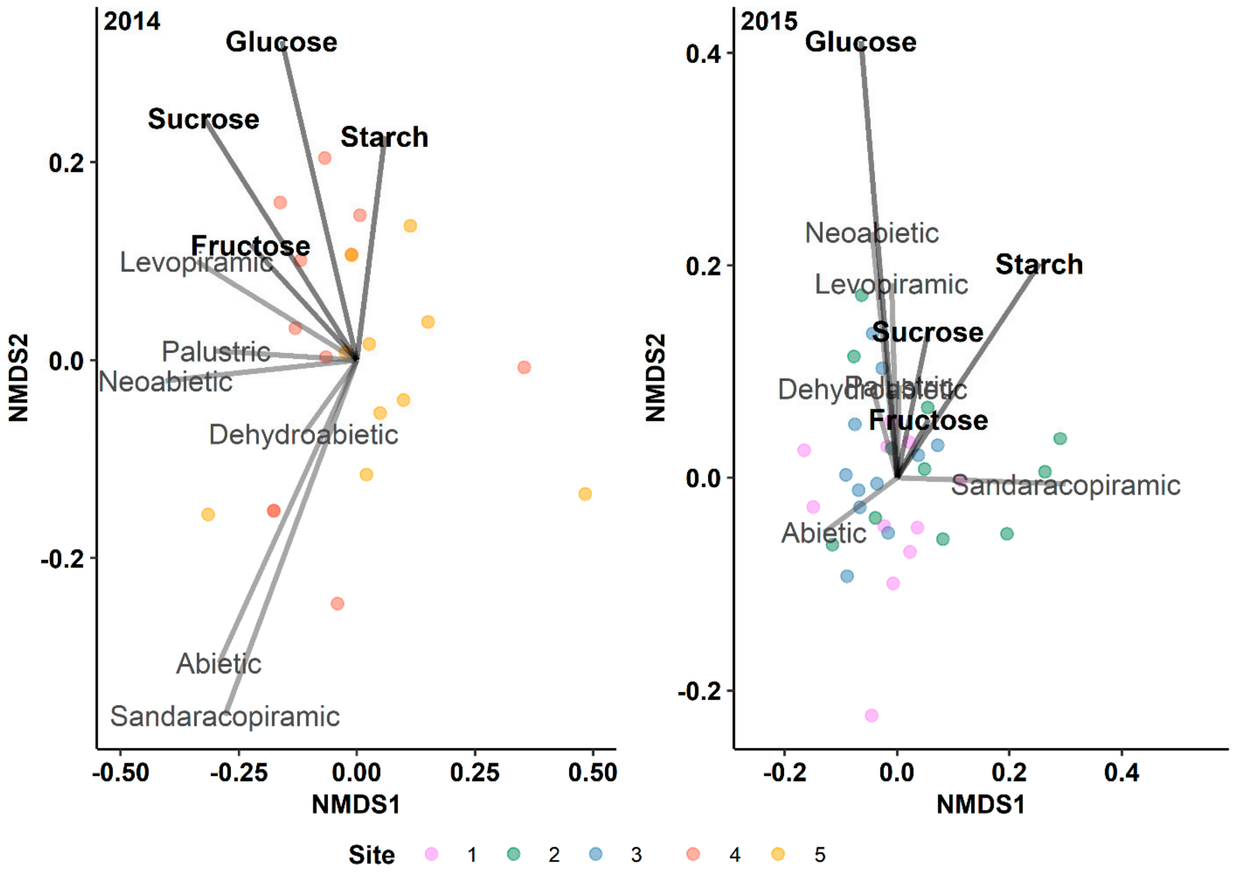

Figure S2. Results of indirect gradient analysis by NMDS with Bray–Curtis dissimilarity showing the relationship between diterpenes and non-structural carbohydrates of lodgepole pine trees in Jasper National Park (Alberta, Canada) in the 2014 and 2015 outbreak years.

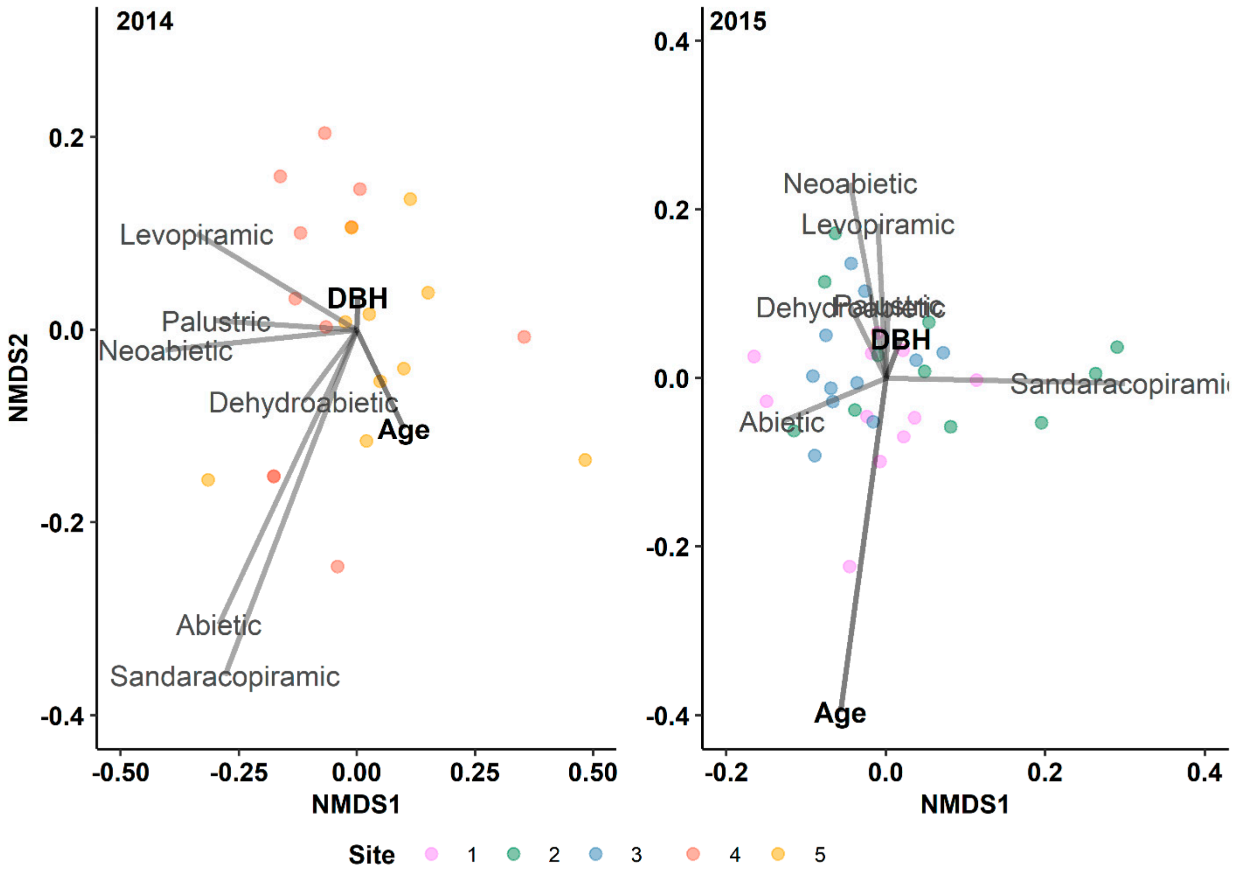

Figure S3. Results of indirect gradient analysis by NMDS with Bray–Curtis dissimilarity showing the relationship between individual diterpenes and diameter at breast height (DBH) and age of lodgepole pine trees in Jasper National Park (Alberta, Canada) in the 2014 and 2015 outbreak years.

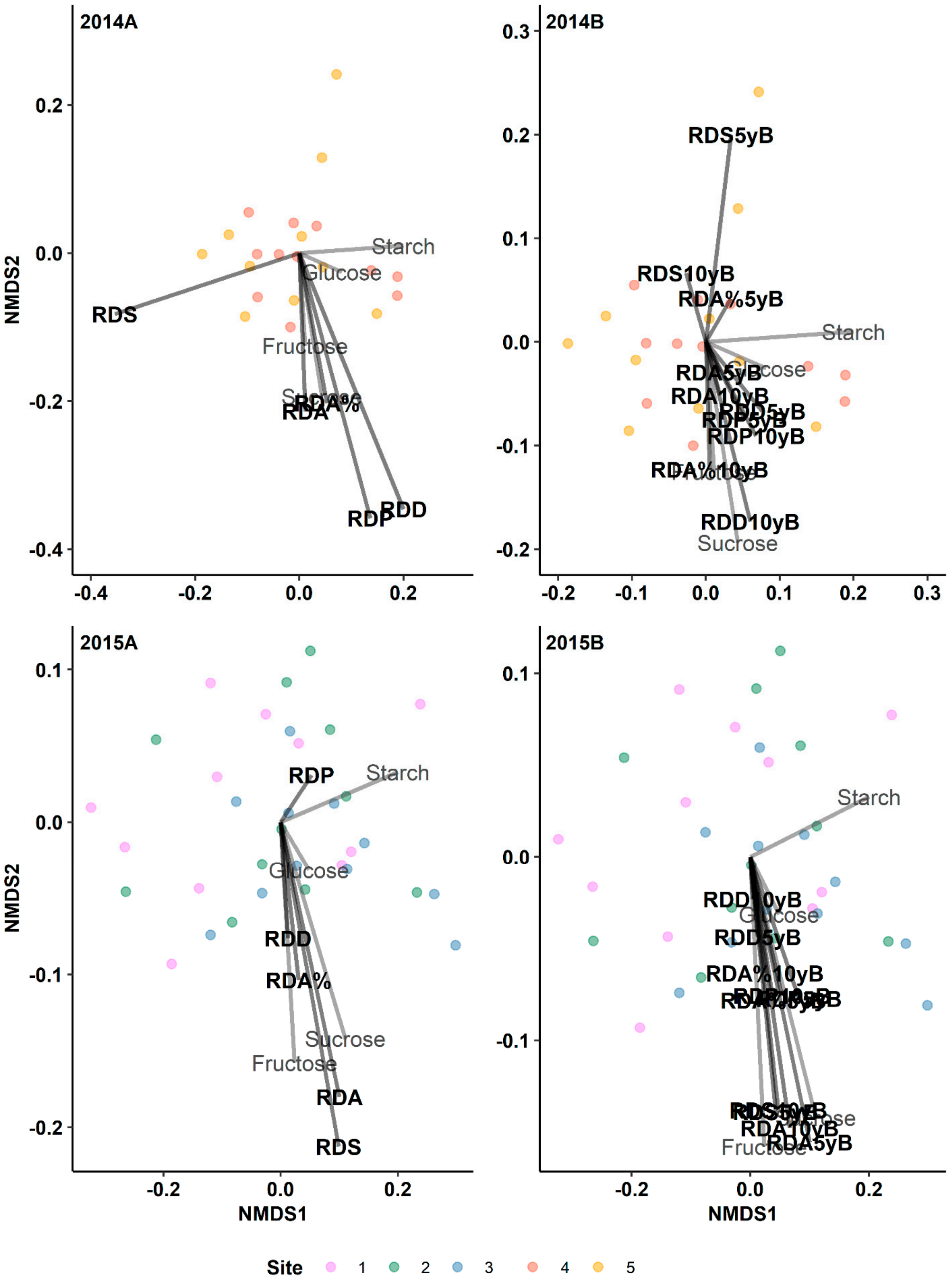

Figure S4. Results of indirect gradient analysis by NMDS with Bray–Curtis dissimilarity showing the relationship between non-structural carbohydrates and each of the five annual resin duct characteristics over post-outbreak **(2014A, 2015A)** and 5-year and 10-year pre-outbreak **(2014B, 2015B)** of lodgepole pine trees in Jasper National Park (Alberta, Canada). The outbreaks occurred in 2014 or 2015. RDP: Resin duct production; RDA: Total resin duct area; RDS: Individual resin duct size; RDD: Resin duct density; RDA%: Relative resin duct area; 5yB: 5-year pre-outbreak; 10yB: 10-year pre-outbreak.

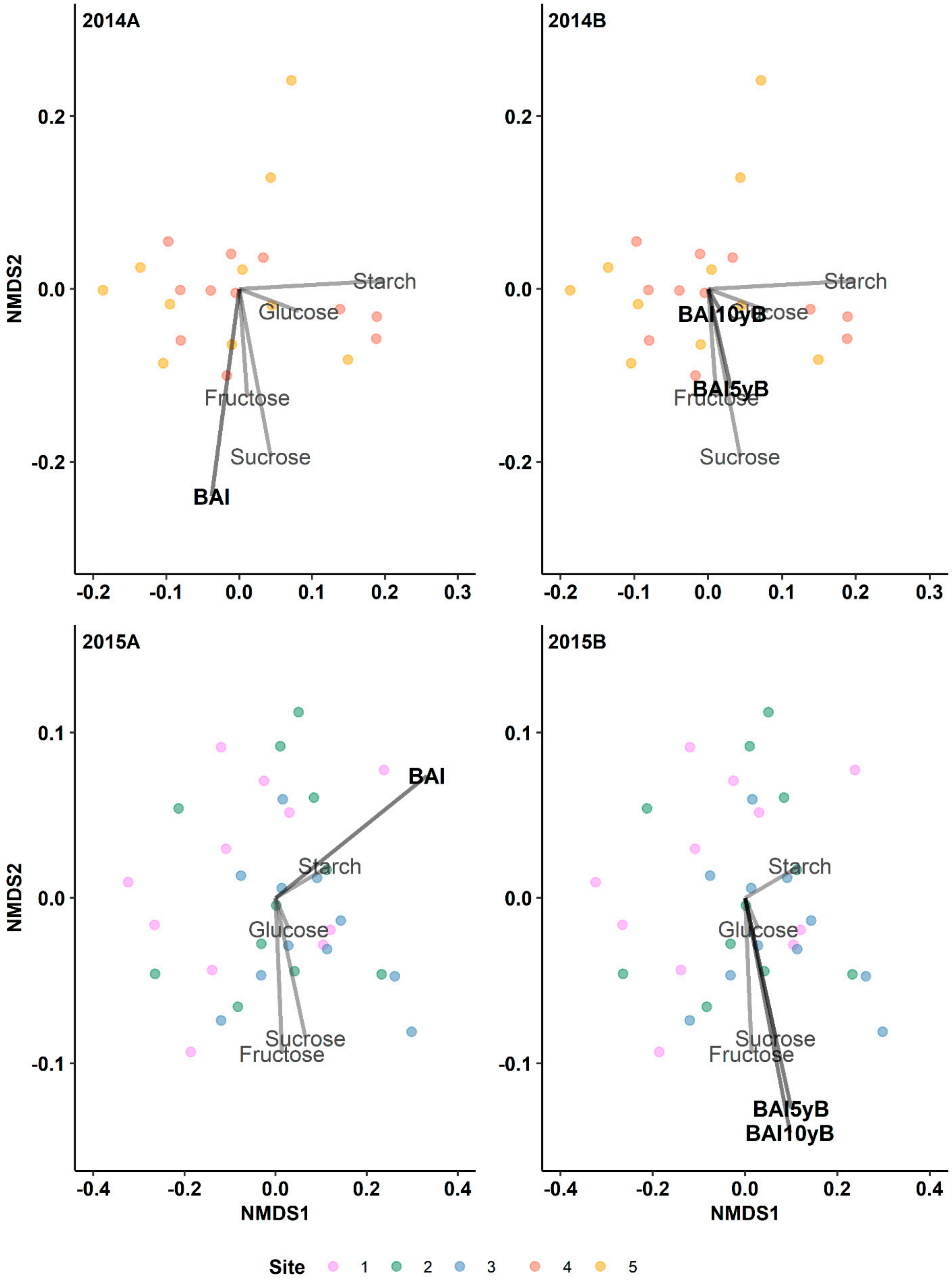

Figure S5. Results of indirect gradient analysis by NMDS with Bray–Curtis dissimilarity showing the relationship between non-structural carbohydrates and the annual basal area increment (BAI, mm^2 $year^{-1}$) over post-outbreak **(2014A, 2015A)** and 5-year and 10-year pre-outbreak **(2014B, 2015B)** of lodgepole pine trees in Jasper National Park (Alberta, Canada) in the 2014 and 2015 outbreak years. 5yB: 5-year pre-outbreak; 10yB: 10-year pre-outbreak.

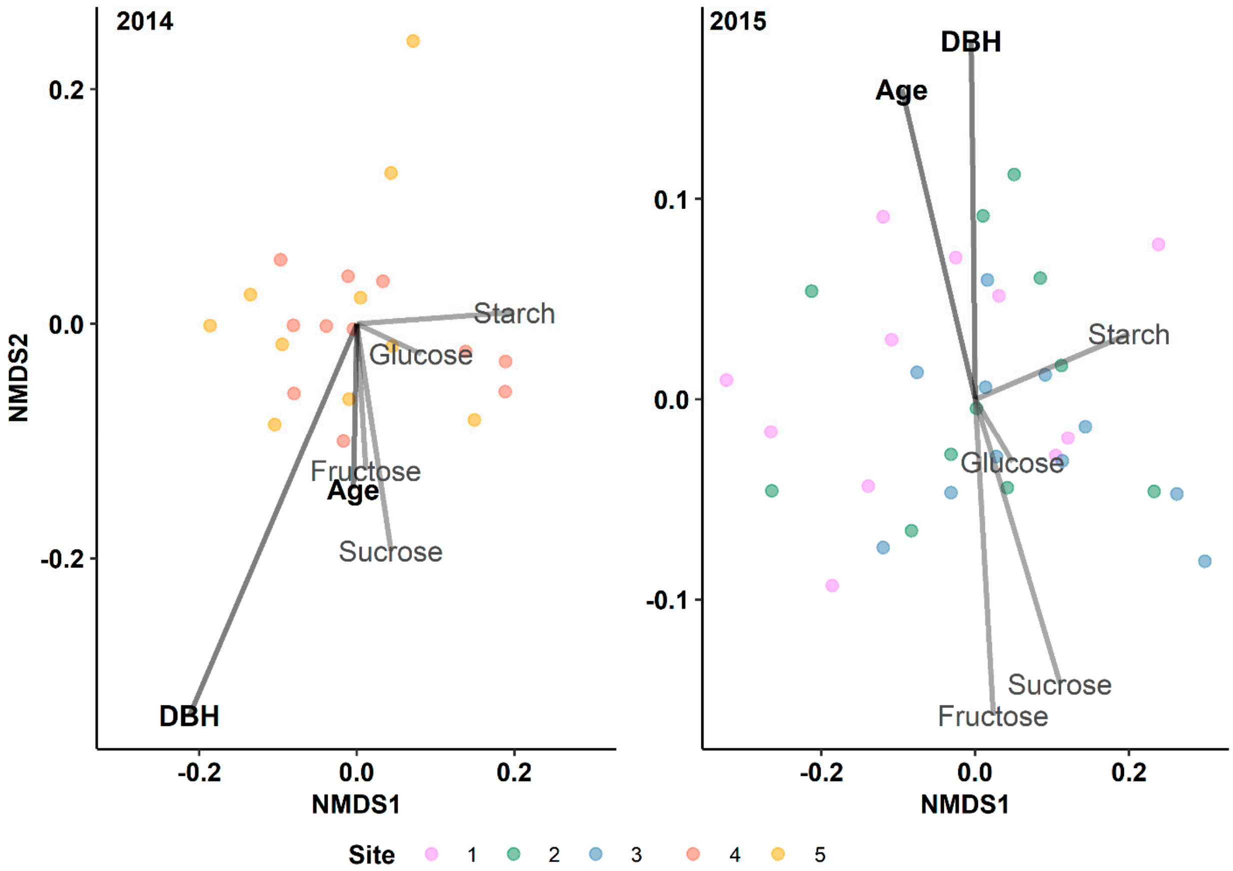

Figure S6. Results of indirect gradient analysis by NMDS with Bray–Curtis dissimilarity showing the relationship between non-structural carbohydrates and diameter at breast height (DBH) and age of lodgepole pine trees in Jasper National Park (Alberta, Canada) in the 2014 and 2015 outbreak years.

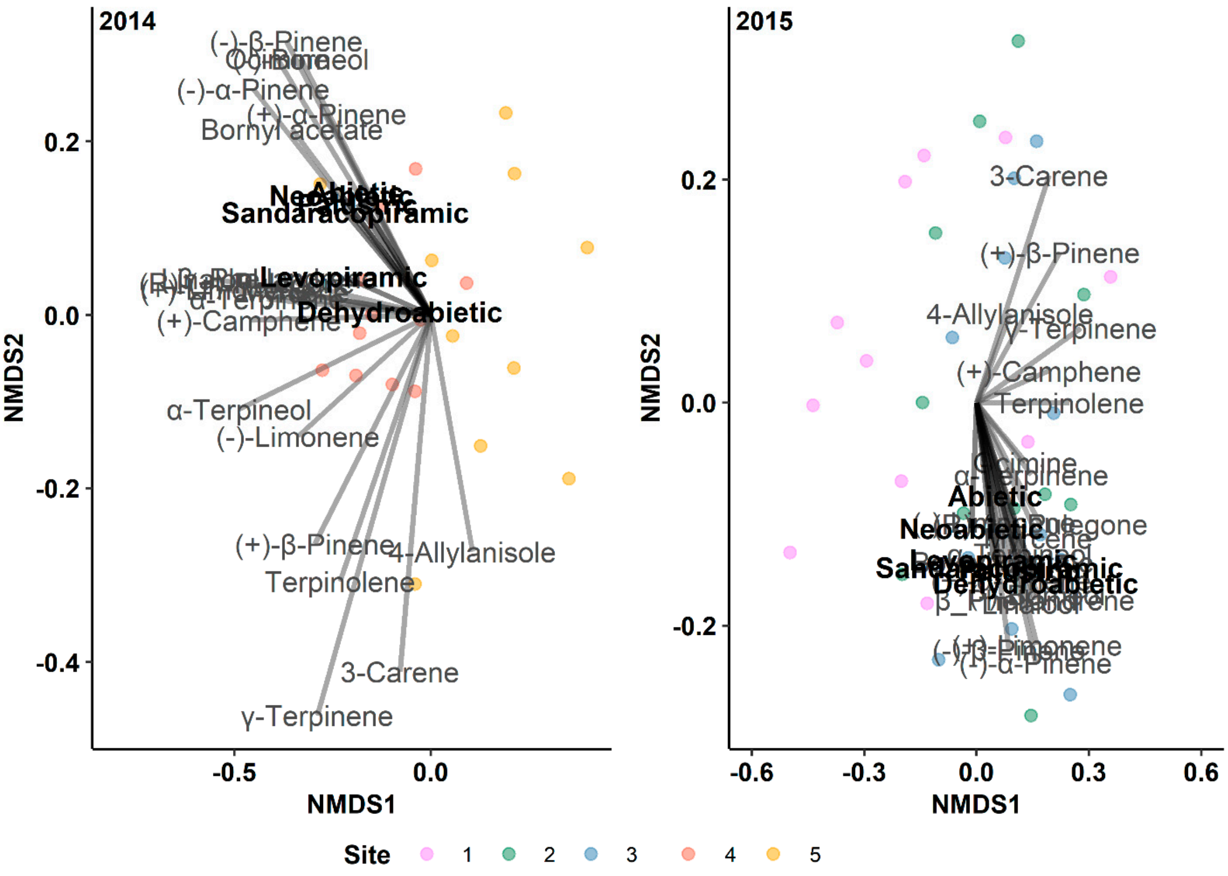

Figure S7. Results of indirect gradient analysis by NMDS with Bray–Curtis dissimilarity showing the relationship between monoterpenes and diterpenes of lodgepole pine trees in Jasper National Park (Alberta, Canada) in the 2014 and 2015 outbreak years.
